# DE-PASS best evidence statement (BESt): determinants of adolescents’ device-based physical activity and sedentary behaviour in settings: a systematic review and meta-analysis

**DOI:** 10.1186/s12889-024-19136-y

**Published:** 2024-06-26

**Authors:** Athanasios Kolovelonis, Ioannis Syrmpas, Anna Marcuzzi, Mohammed Khudair, Kwok Ng, Gavin Daniel Tempest, Ratko Peric, František Bartoš, Maximilian Maier, Mirko Brandes, Angela Carlin, Simone Ciaccioni, Cristina Cortis, Chiara Corvino, Andrea Di Credico, Patrik Drid, Francesca Gallè, Pascal Izzicupo, Henriette Jahre, Atle Kongsvold, Evangelia Kouidi, Paul Jarle Mork, Federico Palumbo, Penny Louise Sheena Rumbold, Petru Sandu, Mette Stavnsbo, Sofia Vilela, Catherine Woods, Kathrin Wunsch, Laura Capranica, Ciaran MacDonncha, Fiona Chun Man Ling, on behalf of DE-PASS

**Affiliations:** 1 Department of Physical Education and Sport Science, University of Thessaly, 42 100 Karies, Trikala, Greece; 2 Department of Public Health and Nursing, Norwegian University of Science and Technology (NTNU), Trondheim, Norway; 3 Department of Sport, Exercise and Rehabilitation, Northumbria University, Newcastle, UK; 4 Faculty of Education, University of Turku, Turku, Finland; 5 Department of Physical Education and Sport Sciences, Physical Activity for Health Centre, University of Limerick, Limerick, Ireland; 6 Institute of Innovation and Sports Science, Lithuanian Sports University, Kaunas, Lithuania; 7 Exercise Physiology Laboratory, OrthoSport Banja Luka, Banja Luka, Bosnia-Herzegovina; 8 Department of Psychological Methods, University of Amsterdam, Amsterdam, the Netherlands; 9 University College London, London, UK; 10 Department of Prevention and Evaluation, Leibniz, Institute for Prevention Research and Epidemiology – BIPS, Bremen, Germany; 11 Centre for Exercise Medicine, Physical Activity and Health, Sport and Exercise Sciences Research Institute, Ulster University, Belfast, UK; 12 Department of Movement, Human and Health Sciences, University of Rome “Foro Italico”, Rome, Italy; 13 Department of Human Sciences, Society and Health, University of Cassino and Lazio Meridionale, Cassino, Italy; 14 Faculty of Economics, Department of Psychology, Universita Cattolica del Sacro Cuore, Milan, Italy; 15 Department of Medicine and Aging Sciences, University “G. d’Annunzio” of Chieti-Pescara, Chieti, Italy; 16 Faculty of Sport and Physical Education, University of Novi Sad, Novi Sad, Serbia; 17 Department of Medical, Movement and Wellbeing Sciences, University of Naples “Parthenope”, Naples, Italy; 18 Department of Rehabilitation Science and Health Technology, Oslo Metropolitan University, Oslo, Norway; 19 Laboratory of Sports Medicne, Department of Physical Education and Sports Science, Aristotle University of Thessaloniki, Thessaloniki, Greece; 20 Health Promotion and Evaluation, National Institute of Public Health in Romania, Bucharest, Romania; 21 Department of Sport Science and Physical Education, Faculty of Health and Sport Sciences, University of Agder, Kristiansand, Norway; 22 EPIUnit - Institute of Public Health, University of Porto, Porto, Portugal; 23 Physical Activity for Health Cluster, Department of Physical Education and Sport Sciences, Health Research Institute, University of Limerick, Limerick, Ireland; 24 Institute of Sports and Sports Science, Karlsruhe Institute of Technology, Karlsruhe, Germany

**Keywords:** Physical activity, Adolescents, Robust Bayesian Meta-analysis, Risk of bias, GRADE

## Abstract

**Background:**

Although physical activity (PA) is associated with significant health benefits, only a small percentage of adolescents meet recommended PA levels. This systematic review with meta-analysis explored the modifiable determinants of adolescents’ device-based PA and/or sedentary behaviour (SB), evaluated in previous interventions and examined the associations between PA/SB and these determinants in settings.

**Methods:**

A search was conducted on five electronic databases, including papers published from January 2010 to July 2023. Randomized Controlled Trials (RCTs) or Controlled Trials (CTs) measuring adolescents’ device-based PA/SB and their modifiable determinants at least at two time points: pre- and post-intervention were considered eligible. PA/SB and determinants were the main outcomes. Modifiable determinants were classified after data extraction adopting the social-ecological perspective. Robust Bayesian meta-analyses (RoBMA) were performed per each study setting. Outcomes identified in only one study were presented narratively. The risk of bias for each study and the certainty of the evidence for each meta-analysis were evaluated. The publication bias was also checked. PROSPERO ID: CRD42021282874.

**Results:**

Fourteen RCTs (eight in school, three in school and family, and one in the family setting) and one CT (in the school setting) were included. Fifty-four modifiable determinants were identified and were combined into 33 broader determinants (21 individual–psychological, four individual–behavioural, seven interpersonal, and one institutional). RoBMAs revealed none or negligible pooled intervention effects on PA/SB or determinants in all settings. The certainty of the evidence of the impact of interventions on outcomes ranged from very low to low. Narratively, intervention effects in favour of the experimental group were detected in school setting for the determinants: knowledge of the environment for practicing PA, *d* = 1.84, 95%CI (1.48, 2.20), behaviour change techniques, *d* = 0.90, 95%CI (0.09, 1.70), choice provided, *d* = 0.70, 95%CI (0.36, 1.03), but no corresponding effects on PA or SB were found.

**Conclusions:**

Weak to minimal evidence regarding the associations between the identified modifiable determinants and adolescents’ device-based PA/SB in settings were found, probably due to intervention ineffectiveness. Well-designed and well-implemented multicomponent interventions should further explore the variety of modifiable determinants of adolescents’ PA/SB, including policy and environmental variables.

**Supplementary Information:**

The online version contains supplementary material available at 10.1186/s12889-024-19136-y.

## Background

Regular physical activity (PA) is associated with significant health-related benefits [1], effective cognitive functioning [2], and higher academic performance [3]. Conversely, physical inactivity is associated with an increased prevalence of obesity and cardiovascular diseases [1, 4]. Thus, increasing PA levels can be considered a cost-effective strategy for improving people’s health and reducing the burden on health-care systems [5]. Considering this evidence, the World Health Organization (WHO) [6] recommends that children and adolescents should partake in at least 60 min of moderate to vigorous PA (MVPA) every day. However, globally, only one out of five adolescents meet WHO’s recommended levels of PA [4, 7, 8]. Moreover, the WHO has suggested that adolescents should reduce sedentary behaviours (SB), especially recreational screen time [4, 6]. However, adolescents spend a lot of their leisure time in SB (e.g., screen-viewing) which has been associated with unfavourable body composition, lower fitness, and lower self-esteem [9]. The SB prevalence in European adolescents (boys and girls) seems to be extremely high (e.g., 76.8% in 2017) [10]. Other evidence has suggested that the average screen time for children and adolescents was 2.9 h/day while the total SB was 8.1 h/day and increased from early childhood through adolescence [11].

To reverse this alarming trend, the WHO has set a goal of reducing the incidence of worldwide physical inactivity by 15% by 2030 [12]. This goal can only be achieved if effective policies aimed at promoting PA and reducing SB are implemented [12, 13]. The European Union has also emphasized the need to implement effective policies to promote health-enhancing PA [14]. Policies provide the framework within which programmes and environmental interventions can operate [15] and as such they should be based on high-quality research evidence regarding the factors associated with adolescents’ PA in different settings. Understanding which drivers (i.e., modifiable determinants) of PA work effectively in the various social or environmental contexts (i.e., settings), how these determinants interact with each other, and how to incorporate them in well-organized systems is critical for designing effective PA interventions [13, 16].

In this context, research should focus on understanding the determinants of PA/SB in different settings. Determinants can be viewed as causal factors and mechanisms that include personal, social, economic, and environmental factors that drive and explain adaptations of behaviour in specific contexts [17]. From a social-ecological perspective, determinants can be individual (e.g., psychological, behavioural), interpersonal (e.g., relationships with parents or peers), but also institutional, environmental (e.g., organization or neighborhood characteristics), community, or policy-related (e.g., laws, policies) [18]. Determinants can be non-modifiable (e.g., age, gender) or modifiable (e.g., motivation, self-efficacy, family support, or transport infrastructure) meaning that they could be altered through an intervention [17]. Moreover, adolescents face rapid psychological and biological changes while being influenced by various determinants in different settings (e.g., school, family, neighborhood, and social networks). Hence, it is useful for researchers to identify which of these determinants have a positive impact on adolescents’ PA to design and implement effective interventions and policies to promote PA and to reduce SB [19–21].

The present study is part of the COST Action CA19101 DEterminants of Physical Activities in SettingS (DE-PASS) [https://depass.eu/] that aims to generate a best-evidence statement derived from high-quality research, to inform future interventions and policies targeting PA and SB. To achieve this objective, a series of systematic reviews and meta-analyses (SRMA) were conducted within DE-PASS, to examine the effects of modifiable determinants in promoting PA and/or reducing SB in children and adolescents, in different settings. The present study focused on adolescents’ device-based PA/SB and modifiable determinants in different settings. Device-based measurement methods of PA/SB are considered more sensitive to behaviour change (i.e., alteration in adolescents’ PA/SB) and less susceptible to recall errors [22, 23]. Moreover, by focusing only in device-based measurement methods of PA/SB the results across studies are more comparable and interpretable.

Previous evidence from systematic reviews regarding the effectiveness of determinants on PA/SB [21, 24–30] is mixed and/or inconclusive primarily due to the moderate methodological quality (e.g., lack of assessment for publication bias) and the variety in the research designs and methodologies used (e.g., for measuring PA) in the included studies. In particular, most of the included studies involved a cross-sectional design [26–28] making it difficult to infer causal relationships between determinants and PA/SB. For detecting potential causality between determinants and PA/SB appropriate research designs are needed such as randomized controlled trial (RCT) or controlled trial (CT) [17]. Moreover, some previous reviews focused on mixed populations including both children and adolescents or adults [28, 31, 32] and did not consider the setting of the interventions. Finally, most of the included studies used non-objective measurement methods of PA/SB [25, 27] while some others a combination of self-report and device-based methods [28, 31, 32]. Using different methods for measuring PA/SB may increase methodological variability making the comparison of the results more difficult.

Therefore, the present SRMA expanded previous research efforts by focusing on high-level evidence derived from RCTs or CTs and device-based methods for measuring adolescents’ PA/SB in different settings. Actually, to our knowledge, this is the first SRMA of RCTs and CTs that examined concurrently intervention effects both on modifiable determinants and adolescents’ device-based PA/SB in different settings in order to infer, if possible, potential associations between determinants and PA/SB.

Despite the increasing research interest on adolescents’ PA, further research is needed to enrich our knowledge regarding adolescents’ PA, including intervention implementation and policy development [8]. Such evidence is considered critical for understanding the reasons for PA decline during adolescence [7, 33] and identifying potential barriers and facilitators of PA/SB [34] to develop and implement effective interventions for promoting adolescents’ PA in different settings and informing related public health policies [8, 21]. Consequently, the results of this study can provide valuable information regarding the modifiable determinants that can increase adolescents’ PA or reduce SB more effectively in different settings.

The aims of this SRMA were a) to identify modifiable determinants of adolescents’ device-based PA/SB that were targeted in RCTs and CTs in different settings, b) to examine the effects of these interventions on PA/SB and modifiable determinants and c) to explore the potential associations of these determinants with adolescents’ PA/SB in different settings.

## Methods

### Protocol and registration

A common protocol for all SRMAs for children and adolescents conducted under the DE-PASS consortium has been registered in the international prospective register of systematic reviews (PROSPERO) on October 12, 2021 with the registration number: CRD42021282874 and subsequently published [35]. The present study was reported according to the Preferred Reporting Items for Systematic Reviews and Meta-Analyses (PRISMA) statement [36].

### Eligibility criteria

Eligible studies had to meet the following inclusion criteria: a) adolescents (13–19 years) with no reported medical conditions that would hinder habitual PA, b) adopt a RCT/CT design with an intervention for promoting PA and/or reducing SB and a control or other comparison group, c) report PA/SB as an outcome measure using device-based methods, d) examine modifiable determinants of PA/SB, e) measure both PA/SB and determinants at least at two-time points: pre- and post-intervention, and g) be published in a peer-reviewed journal after 2010 (following the first publication of PA guidelines by the WHO) [37]. High-quality evidence can be derived from RCTs and CTs that can detect potential causality between modifiable determinants and adolescents’ PA/SB [14]. Device-based measurement methods of PA/SB are considered more sensitive to behaviour change (i.e., alteration in adolescents’ PA/SB) compared to self-report methods, which are susceptible to recall errors and bias [22, 23]. All forms of PA were eligible, including structured PA (e.g., PA in physical education), exercise (e.g., gym), leisure-time PA, competitive sport (e.g., football training), active transport PA, or other PA types (e.g., habitual PA). Similarly, various SB activities were included such as screen-based activities (e.g., TV viewing time, homework on computers), transport-related SB (e.g., sitting in a bus) or leisure-time SB (e.g., reading). Νo specific criterion was set regarding the length of the intervention. Studies involving participants outside of the age range (13–19 years) were excluded unless they reported data for a subgroup with a mean age within the eligible range. Grey literature (e.g., research reports, conference proceedings, and theses) was excluded. Studies published in languages other than English were included only if a translation could be provided by a member of the research team.

### Search strategy

A search was conducted in the following electronic databases: PsycINFO (EBSCO), MEDLINE (Ovid), Web of Science, Sport Discus, and Cochrane Central Register of Controlled Trials (CENTRAL) from January 2010 (following the first publication of PA guidelines by the WHO [37]) to September 2021. This search was updated in July 2023 [38]. A detailed description of the search terms, Boolean commands, and field indicators are detailed in the protocol paper [35]. The following search terms were relevant and used for this systematic review (SR): a) PA, b) SB, c) RCT, d) CT, e) determinants commonly used in PA research, f) adolescents, and g) device-based PA/SB measurement methods (e.g., accelerometer, pedometer). Synonyms and related terms that are commonly used in PA/SB research for all these search terms were also used.

### Screening process

Members of the review team performed an initial screening using reference management software (EndNote × 9) [39] to exclude records of grey literature and duplicates. The resulting list of studies was uploaded to Covidence [40], an online tool for SRs, which was used by a group of reviewers to review the studies [36]. After this initial phase, the blinded screening process was completed in two consecutive stages, including title and abstract screening and full-text screening. In both stages, each study was screened by two blinded independent reviewers, randomly selected by Covidence. A third reviewer resolved conflicts, if necessary. The evaluation of studies in the title/abstract and full-text screening was based on a decision tree illustrating the criteria for inclusion/exclusion. Reasons for excluding a study at the full-text stage were recorded. The included studies were then checked for duplicate reporting [41].

### Data extraction

Two independent reviewers extracted data from each study using a form created in Covidence. Missing data or clarifications were requested from the corresponding authors, where necessary. Studies with incomplete data were excluded. Conflicts between reviewers regarding the correctness of the extracted data were resolved through online bilateral consensus meetings. The data extracted included the description of the study and the respective intervention, participants’ characteristics, the measures of PA/SB and modifiable determinants, the study time frames, and results [35].

### Risk of bias

Risk of bias was assessed with a modified version of the Cochrane risk of bias tool for randomized trials (RoB V.2.0) and non-randomized studies of intervention (ROBINS-I) [42], whereby the domain concerning the bias in the measurement of the outcome was duplicated to assess both PA/SB and determinant outcomes. Risk of bias assessment was conducted by the same two independent reviewers who extracted the data from the respective studies. A separate, dedicated form was created in Covidence to facilitate this process. Discrepancies between reviewers were resolved by reaching a consensus on the correctness of the assessment with the contribution of a third reviewer, if necessary.

### Data synthesis and statistical analysis

The main outcomes in the present SRMA were adolescents’ PA and/or SB measured with device-based methods (i.e., accelerometers, pedometers, and phone-based pedometer apps) and the modifiable determinants of PA/SB. The total PA/SB throughout the day was used as an outcome. In cases where total PA/SB was not measured in a study, or if multiple PA/SB outcomes were reported, the outcome most representative of habitual PA/SB (e.g., MVPA/day) was used. Modifiable determinants were classified after data extraction by adopting the social-ecological perspective [18]. In cases where similarities among determinants were identified, conceptually-related determinants were combined into broader determinants. For example, the self-determined motivational regulations (i.e., intrinsic motivation and identified regulation) were merged into the determinant of autonomous motivation, while the non-self-determined motivational regulations (i.e., introjected regulation and external motivation) were merged into the determinant of controlled motivation. Moreover, the psychological needs of autonomy, competence, and relatedness were merged into the determinant of basic psychological needs [43]. By the same token, conceptually similar determinants were analyzed together. For example, determinants related to self-efficacy and confidence regarding PA/SB were all considered under the label of the determinant of self-efficacy. For all these cases, composite scores of multiple outcomes were calculated using respective formulas (Additional file 1) suggested by Borenstein et al. [44]. The robustness of the composite scores and the effect sizes, when different correlation coefficients were applied to the calculation, were tested through a sensitivity analysis [44].

Outcomes (i.e., PA/SB and determinants) were included in meta-analysis (MA) by study setting providing that at least two studies reported the same outcome in a specific setting. Intervention effects on determinants were analyzed regardless of their PA/SB outcomes. The results of the outcomes identified in only one study were presented narratively. Studies including habitual and non-habitual PA (e.g., PA during physical education) were analyzed separately. Short-term (up to 6 months) and long-term (over 6 months) follow-up measurements were analyzed separately.

The effect size metric of the “standardised mean difference” and the standard error were calculated for all outcomes of studies included in this SR and meta-analyzed or presented narratively. MAs were performed in JASP 0.17.1 statistics software [45] adopting the robust Bayesian meta-analysis (RoBMA) [46] which uses the RoBMA R package [47] and Markov Chain Monte Carlo algorithms via JAGS [48]. We used only random-effects part of the RoBMA model ensemble with the default prior distributions resulting in 18 included models (detailed RoBMA specification can be found in [49]). We used Bayes factor (BF_10_) to measure evidence of the presence of an effect (alternative hypothesis) over the absence of an effect (null hypothesis). The same criteria were also applied to publication bias assessment. BF_10_ were interpreted using the Lee and Wagenmakers’ thresholds [50]. BF_10_ values between 1 and 0.33 represent anecdotal evidence (i.e., presence or absence of an effect cannot be ascertained), BF_10_ values between 0.33 and 0.1 represent moderate evidence, and BF_10_ values below 0.1 represent strong evidence for the null hypothesis. A detailed presentation of the cut-off criteria for the interpretation of BF_10_ is included in Additional file 2. The effect sizes of Cohen’s d with a 95% credible interval (CI) were also reported. For interpreting these results, effect size values above 0.20 were considered a small effect, values above 0.50 a moderate effect and values above 0.80 a large effect [51]. The degree of heterogeneity was assessed by the between-study standard deviation τ. For readers unfamiliar with RoBMA, classical frequentist MAs with random effects were additionally performed and the results including effect size (95% CI) and heterogeneity are presented in Additional file 3.

### Certainty of evidence

The certainty of the evidence for each outcome was evaluated with the Grading Recommendations to Assess Development and Evaluation system (GRADE) [52]. The GRADE classification includes four possible levels: Very low (the true effect is probably markedly different from the estimated effect); Low (the true effect might be markedly different from the estimated effect); Moderate (the true effect is probably close to the estimated effect); High (the true effect is similar to the estimated effect). Five factors, namely the risk of bias, imprecision, inconsistency, indirectness, and publication bias can be considered relevant for downgrading the certainty of the evidence. Two independent reviewers evaluated the certainty of the evidence using GRADE. Discrepancies between reviewers were resolved by achieving a consensus, while a third reviewer was consulted, if necessary.

### Training process

All reviewers involved in the screening process, data extraction, assessment of the risk of bias and certainty of evidence attended online workshops, to perform the above-described steps efficiently and to ensure mutual understanding and consistent practice. A constant communication process among reviewers and with the study leaders was also maintained during all stages of the SRMA research process.

## Results

### Study selection

**Fig. 1:**
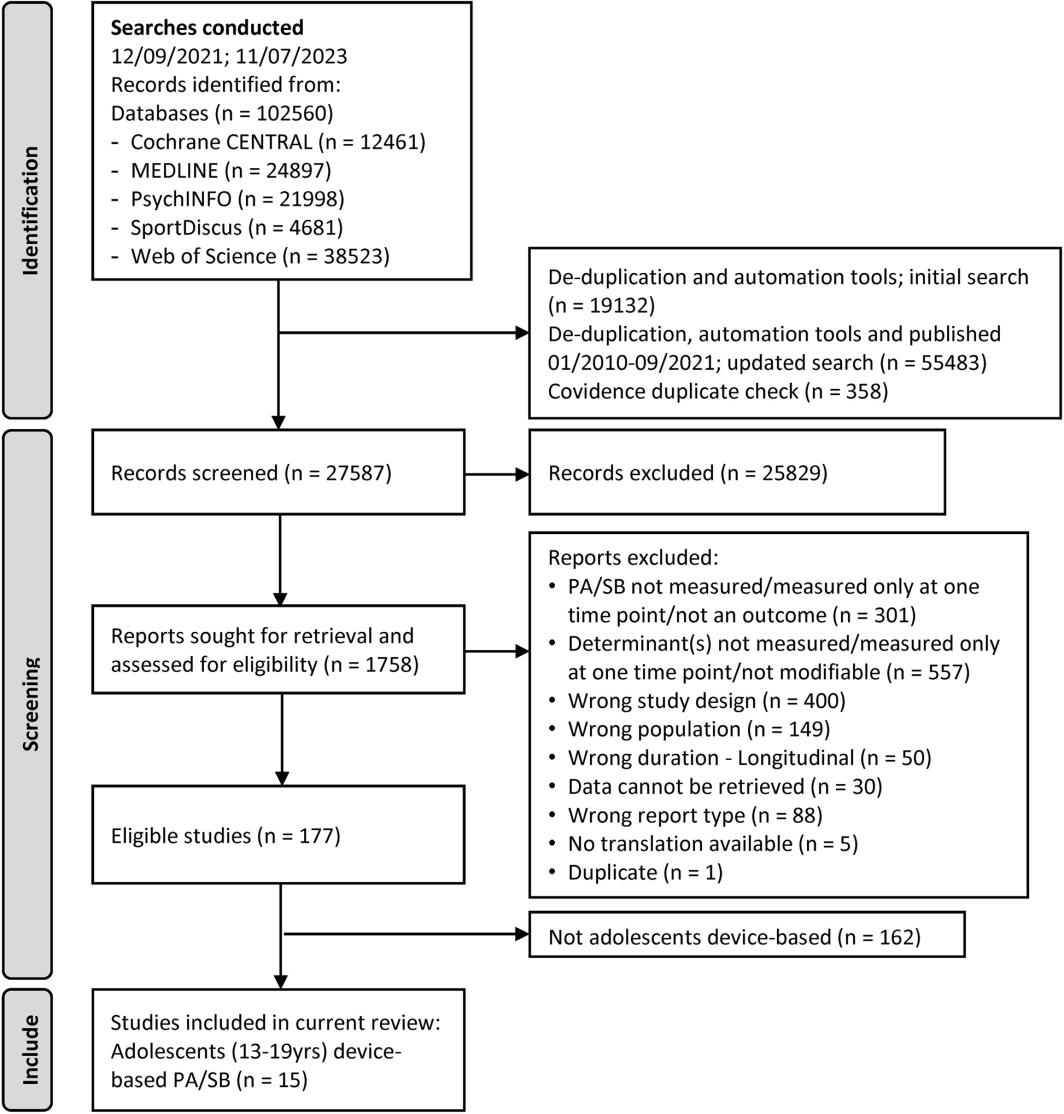
PRISMA flowchart of included studies, eligibility, inclusion, and exclusion criteria

In total, 102,560 records were identified through the search of electronic databases. After removing duplicate results, 27,587 records were included in the screening process. Title and abstract screening resulted in 1,758 full-text articles. Full-text screening resulted in 177 eligible studies for all DE-PASS reviews, focusing on children and adolescents and involving RCT, CT and longitudinal studies. The present review included the 15 studies (14 RCTs, one CT) measuring adolescents’ device-based PA/SB measurement and modifiable determinants (PRISMA flow diagram Fig. 1 [36]).

### Study characteristics

The characteristics of the included studies are summarized in Table 1. A total number of 8531 adolescents (5310 girls) participated in the included studies ranging from 40 to 2862 participants in each of the individual studies.

**Table 1:** Characteristics of the included studies

Study (Country)	Description of Intervention	Intervention duration (follow-up)	Comparison group(s)	Theoretical basis	Sample characteristics at baseline	Type of PA/SB—measurement	Determinant (measurement)	C^a^
**School setting**								
Andruschko et al., 2018 [59] (Australia)	The Sport4Fun intervention consisted of two practical components (a compulsory 90min weekly movement skill activity in school sport and a non-compulsory 60min after school sports-based program) and one theoretical component (three 15min sessions per week during homeroom)	6 months (n/a)	Control – Usual PE curriculum	Social cognitive theory	*N* = 20 Age: 12-15y Mean age (sd): 13.2 (0.9) Girls: 52.5%	Total weekday PA and SB (counts/min) Accelerometer (ActiGraph)	Perceived physical competence (Southall et al., 2004)	1
							Fundamental Movement Skills (NSW Department of Education & Training, 2000)	2
							Enjoyment (Motl et al., 2001)	1
Casado-Robles et al., 2022 [65] (Spain)	Intervention consisted of delivering inside lessons (i.e., in-school teaching unit, using conventional school facilities) followed by outside lessons in the immediate environment (i.e., out-of-school teaching unit, using outside installations and features, green zones, or a municipal sport center)	4 weeks (n/a)	Control – Fitness, traditional and alternative games and sports	Self-determination theory, Trans-contextual model	*N* = 171 Age: 13-15y Mean age (sd): 14.05 (0.95) Girls: 52.5%	%MVPA and %SB Accelerometer (ActiGraph)	Knowledge about the environment to practice PA (Casado-Robles et al., 2021)	1
							Perceived autonomy support (Moreno et al., 2008)	1
							Autonomous and controlled motivation (BREQ-3, González-Cutre et al., 2010)	1
							Intention to be physically active (Granero-Gallegos et al., 2014)	1
Corder et al., 2016 [53] (UK)	Implementation of the GoActive intervention aimed at increasing PA through increased peer support, self-efficacy, self-esteem, group cohesion and friendship quality. The intervention was implemented in tutor groups using a tiered leadership system	8 weeks (n/a)	Control—No intervention	Based on the strengths of various models and approaches	*N* = 460 Age: 13-14y Mean age (sd): 13.2 (0.4) Girls: 46.6%	Average daily minutes in MVPA Accelerometer (ActiGraph)	Self-efficacy for PA (Saunders et al., 1997)	1
							Social support by peers (Ommundsen et al., 2008)	3
							Friendship quality (Goodyer et al., 1997)	3
							Shyness (Buss & Plomin, 1984)	1
							Sociability (Buss & Plomin, 1984)	1
							Barriers to PA (no reference provided)	1
Corder et al., 2020 [54] (UK)	GoActive intervention aimed to maximize students’ PA through increased peer support, self-efficacy, self-esteem, and friendship quality, and was implemented in tutor groups using a student-led tiered-leadership system	12 weeks (10 months)	Control—No intervention	Evidence-based approach, multiple guidelines and frameworks	*N* = 2862 Age 13-14y Mean age (sd): 13.2 (0.4) Girls: 47.9%	MVPA (min/day) SB (min/day) Accelerometer (Axivity)	Self-efficacy for PA (Saunders et al., 1997)	1
							Friendship quality (Goodyer et al., 1997)	3
							Social support by peers (Ommundsen et al., 2008)	3
							Self-esteem (Rosnberg, 1979)	1
Ha et al., 2020 [55] (China)	A Self-determined Exercise and Learning for FITness (SELF-FIT) program aimed to maximise students' MVPA, infuse fitness and game-like elements into PE using self-determination theory principles, and enhance students’ need satisfaction and autonomous motivation	8 lessons (n/a)	Control (wait-list)—Usual practices	Self-determination theory	*N* = 667 Mean age (sd): 14.4 (0.78) Girls: 69%	PA (7-day counts/min) Accelerometer (ActiGraph)	Basic psychological needs (autonomy, competence, relatedness) (Ng et al., 2011)	1
							Autonomous and controlled motivation (PLCQ, Lonsdale et al., 2011)	1
							Perceived need support from teachers (LCQ, Williams et al., 1994)	3
Hankonen et al., 2017 [60] (Finland)	Multi-level intervention (Let’s Move It) aimed to increase PA and decrease SB in vocational school students, consisted of a 6-h group-based intervention for students, two 2-h training for teachers to reduce students’ sitting in class, and provision of light PA equipment in classrooms	6 weeks (n/a)	Control – Usual PE + leaflet on recommendations for youth PA	Self-determination theory, Self-regulation theories	*N* = 40 Age: 17-25y Mean age (sd): 18.9 (1.67) Girls: 85%	%MVPA and %SB (out of total wear time) Accelerometer (Hookie)	BCT (items developed for this study—no reference provided)	2
Jago et al., 2021 [56] (UK)	The PLAN-A (Peer-Led physical Activity iNtervention for Adolescent girls) program focuses on increasing PA in secondary school age girls, providing choice and autonomy over PA while building PA competence and connectedness with others (peer support)	10 weeks (n/a)	Control – Usual PE curriculum	Diffusion of innovation theory, Self-determination theory	*N* = 1558 Age: 13-14y Mean age (sd): 13.80 (0.33) Girls: 100%	Weekday and Weekend MVPA and SB (in minutes) Accelerometer (ActiGraph)	Autonomous and controlled motivation (BREQ-2, Markland & Tobin, 2004)	1
							Basic psychological needs (autonomy, competence, relatedness) (Standage et al., 2004; McAuley et al., 1989)	1
							PA self-efficacy (Bartholomew et al., 2006)	1
							Social support for PA by peers (Mendonça, 2015)	3
							Peer norms of PA: prevalence, importance, acceptance (no reference provided)	3
Lonsdale et al., 2013 [57] (Australia)	Three intervention groups were included: (1) Relevance group (teachers explained the rationale and importance of the activity to the students' lives); (2) Providing choice group (teachers provided students with 2–4 opportunities for choice within the lesson), and (3) Free choice group (teachers provided the students with equipment but refraining from giving instructions)	1 session (n/a)	Control—Usual teaching practice	Self-determination theory	*N* = 288 Mean age: 13.6 Girls: 49.6%	%MVPA and %SB in a single PE lesson Accelerometer (ActiGraph)	Basic psychological needs: Autonomy (Ng et al., 2011; Standage & Gillison, 2007), Competence (McAuley et al., 1989), Relatedness (Richer & Vallerand, 1998)	1
							Self-determination index (Situational Motivation Scale, Guay et al., 2000; Lonsdale et al., 2009)	1
							Teacher autonomy support: Choice provided, Relevance explained (Taylor & Lonsdale, 2010)	1
Schnider et al., 2021 [58] (Switzerland)	Behavioural skill training as part of compulsory PE aimed to increase adolescents’ PA, exercise/sport intention, motivation, coping planning and self-efficacy. The intervention included three 20-min sessions carried out during a 90-min double PE lesson and focusing on goal setting, implementation intentions and exercise/sport plans in classroom	6 weeks (3 months)	Control – Regular PE (one 45-min and one 90-min lesson per week)	Behavioural (or self-regulatory) skill training	*N* = 128 Age: 14-19y Mean age (sd): 15.8 (1.2) Girls: 52%	PA (steps/day) Accelerometer (ActiGraph)	Motivation: Intrinsic, Identified, Introjected, Extrinsic (Seelig & Fuchs, 2006)	1
							Exercise/sport intention (Seelig & Fuchs, 2006)	1
							Implementation intentions (Sniehotta et al., 2005)	1
							Coping planning (Sniehotta et al., 2005)	1
							Self-efficacy (for barrier coping) (Krämer & Fuchs, 2010)	1
Sudholz et al., 2023 [67]^b^ (Australia)	Height-adjustable desks in combination with prompts (posters and desk stickers) to break up prolonged sitting time during class time. Teachers received professional development in the use of the desks and prompts	17 weeks (n/a)	Control—No intervention	Capability, Opportunity, Motivation, Behavior model	*N* = 105 Age: 12-17y Mean age (sd): 14.8 (1.7) Girls: 43.1%	SB (sitting min/lesson) Accelerometer (activPAL3C)	Habit strength (Gardner et al., 2012)	2
							Self-efficacy (for replacing classroom sitting with standing) ((items developed for this study based on Maher et al., 2012)	1
Verswijveren et al., 2022 [66] (Australia)	The RAW-PA was a multicomponent intervention for adolescents living in areas of socioeconomic disadvantage integrating more physical activity into their day by combining an activity tracker with digital resources that specifically targeted evidence-based behavior-change techniques (e.g., infographics, videos, and social forums)	12 weeks (6 months)	Control (wait-list) – No intervention	Social cognitive theory, Behavioral choice theory	*N* = 159 Mean age (sd): 13.7 (0.4) Girls: 58.5%	Average daily MVPA and sedentary time Accelerometer (ActiGraph)	Self-efficacy (Dewar et al., 2013)	1
							Peer support (Dewar et al., 2013)	3
							Family support (Dewar et al., 2013)	3
							Teacher support (Dewar et al., 2013)	3
							Self-regulation strategies (Dewar et al., 2013)	1
							Perceived barriers to PA (Robbins et al., 2008)	1
							Enjoyment (Motl et al., 2001)	1
**School and family setting**								
Aittasalo et al., 2019 [61] (Finland)	The Kids Out! intervention integrating behavioral theory-driven content into three routinely scheduled HE lessons in secondary schools. The multimodal content included Internet-based self-assessment with feedback views, YouTube-video, refillable student leaflet, refillable classroom poster, classroom peer-discussions and parental leaflet for influencing their children's PA	4 weeks (n/a)	Control – Standard HE lessons on PA	Health action process approach model	*N* = 1550 Mean age (sd): 13.9 (0.5) Girls: 47.8%	Total PA (min/day) Accelerometer (Hookie)	Short-term behavioural intention (related to walking/cycling to school, leisure PA, and screen time) (Roberts et al., 2010)	1
							Confidence to execute behavioural intention (related to walking/cycling to school, leisure PA, and screen time) (Roberts et al., 2010)	1
							Family norm (items developed for this study—no reference provided)	3
Dewar et al., 2014 [62] (Australia)	The Nutrition and Enjoyable Activity for Teen Girls (NEAT Girls) program was a school-based intervention designed to prevent unhealthy weight gain in adolescent girls living in low-income communities through improving PA, dietary and reducing SB. The intervention included enhanced school sports sessions (40 × 90 min), interactive seminars, student handbooks, nutrition workshops, parent newsletters and text messages	12 months (n/a)	Control – No intervention	Social cognitive theory	*N* = 357 Mean age (sd): 13.2 (0.5) Girls: 100%	Total MVPA (%) and total SB (%) Accelerometer (ActiGraph)	Self-efficacy (Dewar et al., 2012)	1
							Perceived environment (home/ neighborhood) (Dewar et al., 2012)	4
							Perceived environment (school) (Dewar et al., 2012)	4
							Social support by friends (Dewar et al., 2012)	3
							Social support by family (Dewar et al., 2012)	3
							Behavioral strategies (Dewar et al., 2012)	2
							Outcome expectations (Dewar et al., 2012)	1
							Outcome expectancies (Dewar et al., 2012)	1
							Self-efficacy (Motl et al., 2000)	1
Lubans et al., 2010 [63] (Australia)	Extra-curricular school sport program (Program X) involving: (1) enhanced school sport program focusing on lifetime PA, (2) information sessions focusing on PA and healthy eating, (3) pedometers for PA monitoring, (4) PA and nutrition handbooks and monthly information newsletters for parents, (5) social support (by e-mail) for healthy behaviors	10 weeks (n/a)	Control—the 10-week school sport without the strategies for behavior change	Social cognitive theory	*N* = 124 Mean age (sd): 14.1 (0.8) Girls: 57.3%	PA (steps/day) Pedometer	Self-efficacy (Motl et al., 2000)	1
							Family support (Sallis et al., 2002)	3
							Self-management strategies (Saelens et al., 2000)	1
**Family setting**								
Cowley et al., 2021 [64] (UK and Ireland)	The home-based multi-component PA intervention (HERizon Project) for adolescent girls included: (1) Three 30min virtual exercise sessions each week, (2) Behaviour-change support calls (“Activity Mentor”), (3) No-reply sms (three text messages per week aimed at providing PA-related facts, encouragement, and study information)	6 weeks (n/a)	Control (wait-list) – No intervention	Self-determination theory	*N* = 42 Age: 13-16y Mean age (sd): 14.2 (1.1) Girls: 100%	PA (steps/day) Mobile phone-based pedometer app (Google fit)	Motivation: Intrinsic, integrated, Identified, Introjected, Extrinsic, Amotivation (BREQ-3, Markland & Tobin, 2004; Wilson et al., 2006)	1
							Perceived competence (Walston & Smith, 1995)	1
							Self-esteem (Hafekost et al., 2017)	1
							Body appreciation (Avalos et al., 2005)	1

^a^Determinant category: 1 = Individual–psychological, 2 = Individual–behavioural, 3 = Interpersonal, 4 = Institutional

^b^CT (all other studies are RCTs)

### Settings

The 14 RCTs [53–66] and the one CT [67] included were conducted in three settings, namely school (11 studies), school and family (three studies), and family (one study).

### Determinants

In total, 54 modifiable determinants were identified in the 15 studies. These modifiable determinants were classified following the social-ecological perspective [18]. Most of them were individual–psychological (*n* = 37), some were interpersonal (*n* = 11) and a few were individual–behavioural (*n* = 4) or institutional (*n* = 2). After combining conceptually-related determinants into broader determinants, 33 determinants were introduced in the analyses (21 individual–psychological, four individual–behavioural, seven interpersonal and one institutional). Twelve determinants were identified in two or more studies conducted in a specific setting and were, as mentioned in the methods section, included in the meta-analysis, while the rest, identified in only one study, are presented narratively.

### PA and SB outcomes

Thirteen RCTs [53–56, 58–66] measured habitual PA/SB, one RCT [57] non-habitual/structured PA/SB (i.e., during physical education), while one CT [67] measured SB in the classroom. Thirteen studies used accelerometers [53–62, 65, 66], one study used pedometers [63] and one used a mobile phone-based pedometer app [64].

### School setting

Ten RCTs [53–60, 65, 66] and one CT [67] published from 2013 to 2023 were included. Nine RCTs [53–56, 58–60, 65, 66] measured habitual PA/SB, one [57] measured structured PA/SB (i.e., during physical education), and one CT [67] measured SB in the classroom, all using accelerometers. The number of participants in these studies ranged from 40 to 1558 and the intervention duration ranged from four weeks to six months, except for one study including a single physical education session [57]. Three studies [54, 58, 66] included post-intervention follow-up measures (10-, three-, and six-months post-intervention, respectively). All studies based the design of their interventions on one or more theories and all focused on increasing adolescents’ PA and/or decreasing SB and enhancing related psychosocial variables. These studies were implemented during the school schedule either during physical education or in sport-related school programs [53–58], while some others [59, 60, 65, 66] involved additional extra-curricular activities (e.g., asking students to participate in after school PAs) (Table 1).

### RCTs

#### Determinants

**Fig. 2:**
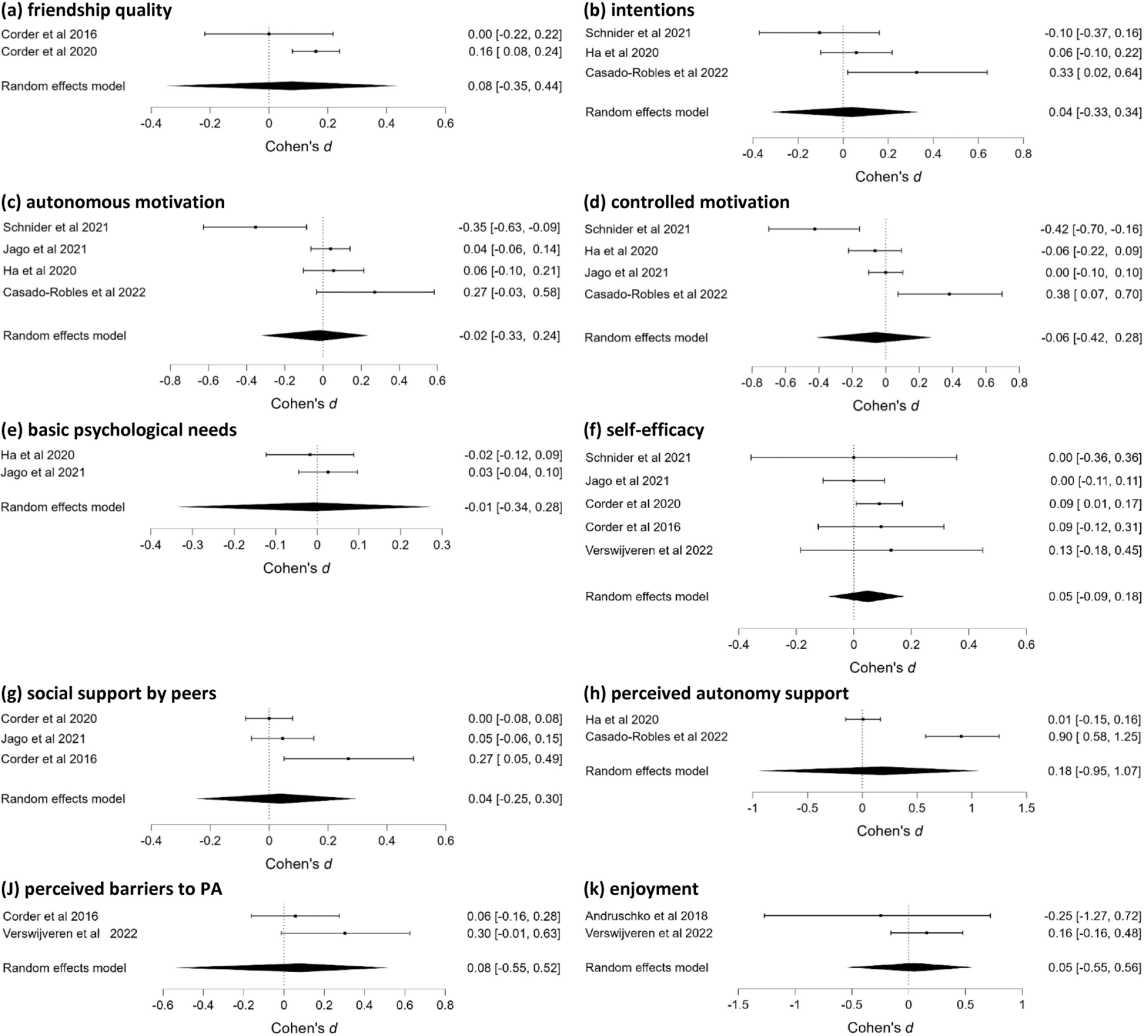
Forest plots of effects of interventions on determinants in the school setting

In total, 36 modifiable determinants were identified in the school setting (25 individual–psychological, three individual–behavioural and eight interpersonal). After merging conceptually-related determinants into broader categories, 25 determinants were included in the analyses (16 individual–psychological, three individual–behavioural, and six interpersonal). Ten of these determinants were included in more than two studies and were meta-analyzed (Table 2; Fig. 2a to k). We found strong evidence for the absence of an effect on autonomous motivation, basic psychological needs and self-efficacy, moderate evidence for the absence of an effect on friendship quality, intentions, controlled motivation, social support by peers, perceived barriers to PA and enjoyment, and anecdotal evidence for the absence of an effect on perceived autonomy support.

**Table 2:** Results of RoBMAs in the school setting for PA, SB, their determinants and the associated heterogeneity and publication bias assessments

	**n**	**Effect size estimates (95%CI)**	**BF**_**10**_
**MA – Friendship quality** (Fig. 2a)	2	0.08 (-0.35, 0.44)	0.20^a^
Heterogeneity (τ)		0.18 (0.05, 0.57)	-
Publication bias		-	0.90
**MA – Intentions** (Fig. 2b)	3	0.04 (-0.33, 0.34)	0.14^a^
Heterogeneity (τ)		0.15 (0.04, 0.45)	-
Publication bias			0.98
**MA – Autonomous motivation** (Fig. 2c)	4	-0.02 (-0.33, 0.24)	0.10^b^
Heterogeneity (τ)		0.16 (0.04, 0.43)	-
Publication bias		-	0.63
**MA – Controlled motivation** (Fig. 2d)	4	-0.06 (-0.42, 0.28)	0.14^a^
Heterogeneity (τ)		0.23 (0.05, 0.57)	-
Publication bias		-	0.64
**MA – Basic psychological needs** (Fig. 2e)	2	-0.01 (-0.34, 0.28)	0.08^b^
Heterogeneity (τ)		0.10 (0.03, 0.31)	-
Publication bias		-	0.60
**MA – Self-efficacy** (Fig. 2f)	5	0.05 (-0.09, 0.18)	0.09^b^
Heterogeneity (τ)		0.09 (0.03, 0.20)	-
Publication bias		-	0.73
**MA – Social support by peers** (Fig. 2g)	3	0.04 (-0.25, 0.30)	0.11^a^
Heterogeneity (τ)		0.11 (0.03, 0.34)	-
Publication bias		-	1.39
**MA – Perceived autonomy support** (Fig. 2h)	2	0.18 (-0.95, 1.07)	0.59
Heterogeneity (τ)		0.54 (0.08, 1.75)	-
Publication bias			1.59
**MA – Perceived barriers to PA** (Fig. 2j)	2	0.08 (-0.55, 0.52)	0.23^a^
Heterogeneity (τ)		0.18 (0.04, 0.63)	-
Publication bias			1.28
**MA – Enjoyment** (Fig. 2k)	2	0.05 (-0.55, 0.56)	0.24^a^
Heterogeneity (τ)		0.20 (0.04, 0.75)	-
Publication bias			0.46
**MA—PA (**Fig. 3a)	9	-0.04 (-0.18, 0.10)	0.07^b^
Heterogeneity (τ)		0.11 (0.03, 0.26)	-
Publication bias		-	0.47
**MA—SB** (Fig. 3b)	6	-0.09 (-0.29, 0.11)	0.17^a^
Heterogeneity (τ)		0.14 (0.05, 0.35)	-
Publication bias		-	0.35
**MA – PA follow-up (**Fig. 3c)	2	-0.22 (-0.77, 0.23)	0.37
Heterogeneity (τ)		0.19 (0.04, 0.70)	-
Publication bias		-	0.50

^a^moderate evidence for absence of an effect

^b^strong evidence for absence of an effect

Fifteen different determinants were identified in only one of the studies conducted in the school setting and are presented narratively. Large standardized mean differences in favour of the experimental group were found in knowledge of the environment for practicing PA,* d* = 1.84, 95%CI (1.48, 2.20) [65], in behaviour change techniques (BCTs), *d* = 0.90, 95%CI (0.09, 1.70) [60] and medium differences in choice provided by teachers, *d* = 0.70, 95%CI (0.36, 1.03) [57]. For other determinants nonsignificant intervention effects (*d*s ranging from -0.41 to 0.71) were found. Notably, there were medium differences in fundamental movement skills, *d* = 0.71, 95%CI (-0.19, 1.61) [59], and low differences in coping planning at post-intervention,* d* = 0.30, 95%CI (-0.05, 0.65) and 3-months follow-up, *d* = 0.31, 95%CI (-0.07, 0.69) [58]. However, in these cases, the 95%CIs crossed the threshold.

#### PA and SB

**Fig. 3:**
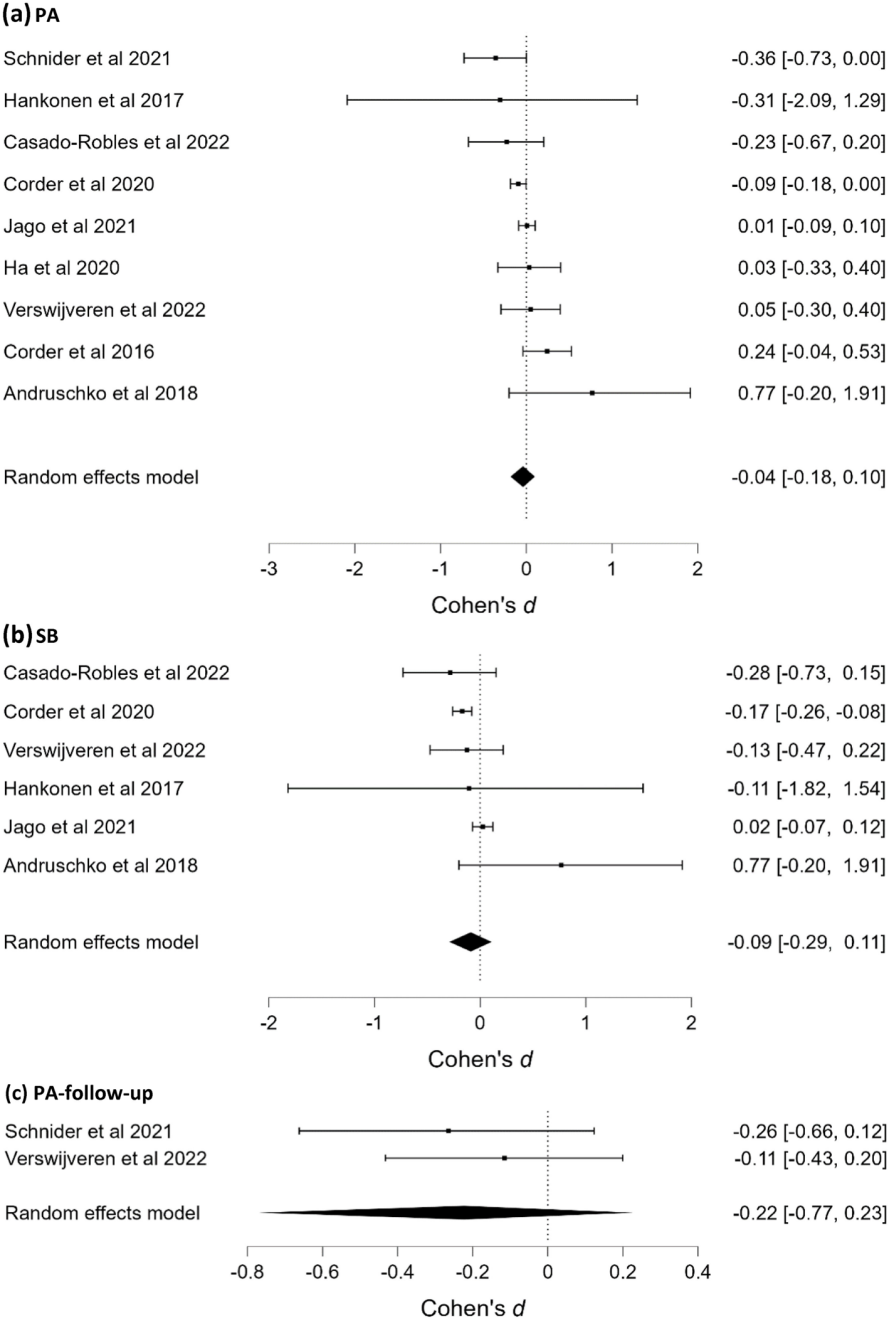
Forest plots of effects of interventions on (**a**) PA, (**b**) SB, and (**c**) on PA-follow-up in the school setting

One RoBMA was conducted for PA (Fig. 3a), one for SB (Fig. 3b) and one for short-term (up to six months) post-intervention follow-up PA (Fig. 3c). Strong evidence for the absence of an effect on PA, moderate evidence for the absence of an effect on SB, and anecdotal evidence for the absence of an effect on follow-up PA were found. One study, included a long-term (over six months) follow-up measure [54] and showed small negative and nonsignificant effects on PA and SB (*d*s = -0.10 and -0.11, respectively). One study [57] measuring non-habitual PA (i.e., MVPA and percentage of SB in a single 20-min physical education session) reported no intervention effects.

All MAs show small heterogeneity accompanied, however, by a large degree of uncertainty due to limited number of studies in each MA. There is also insufficient evidence for publication bias (Table 2).

#### CT

The only CT [67] included in our study, delivered in the school setting, revealed moderate intervention effects, with the 95%CIs crossing the threshold, for habit strength,* d* = 0.44, 95%CI (-0.02, 0.90) and self-efficacy, *d* = 0.39, 95%CI (-0.07, 0.85) for replacing classroom sitting with standing, accompanied with large effects in reducing SB (sitting), *d* = 0.92, 95%CI (0.43, 1.41).

#### Risk of bias assessment

**Fig. 4:**
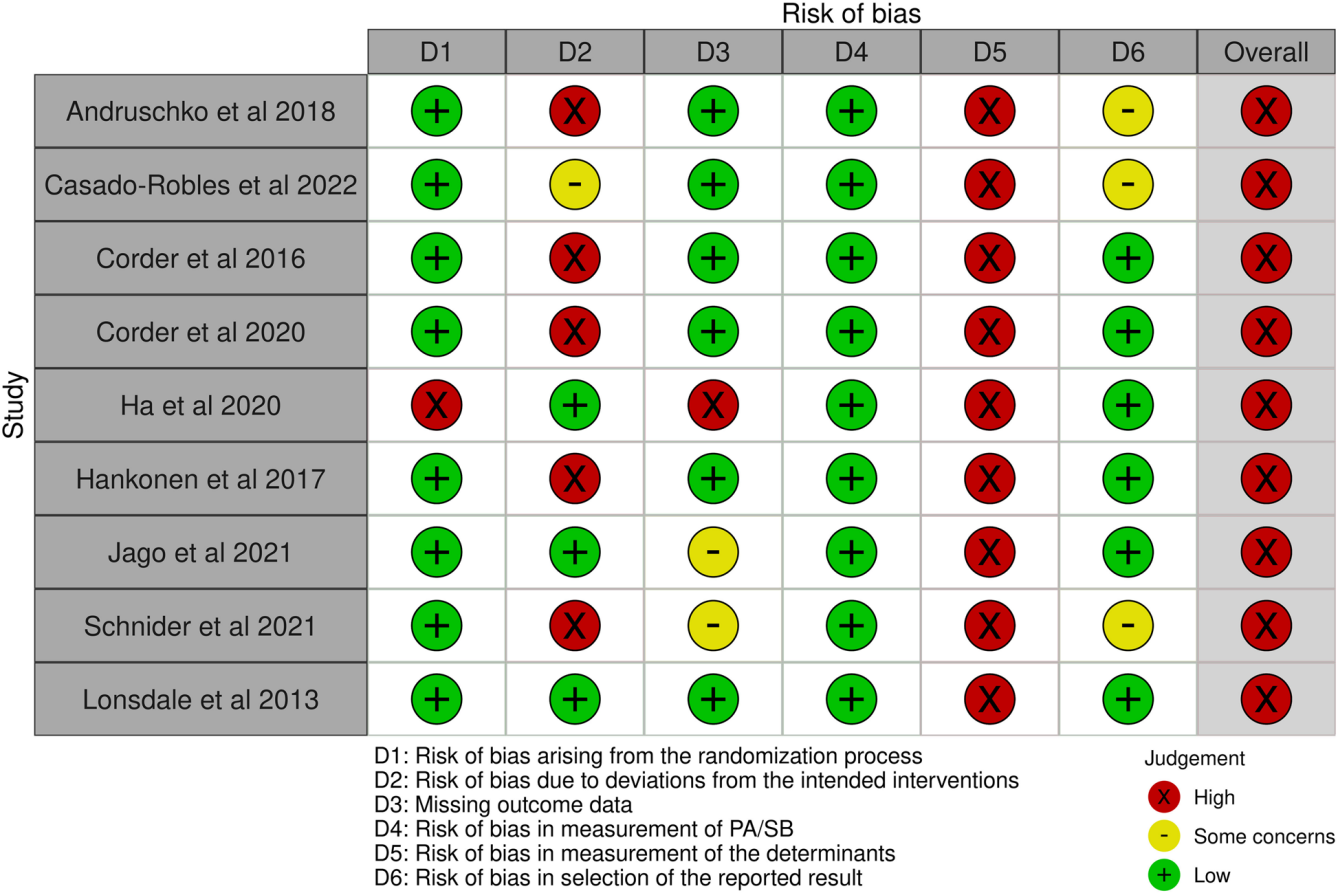
Risk of bias assessments of RCTs in the school setting

**Fig. 5:**
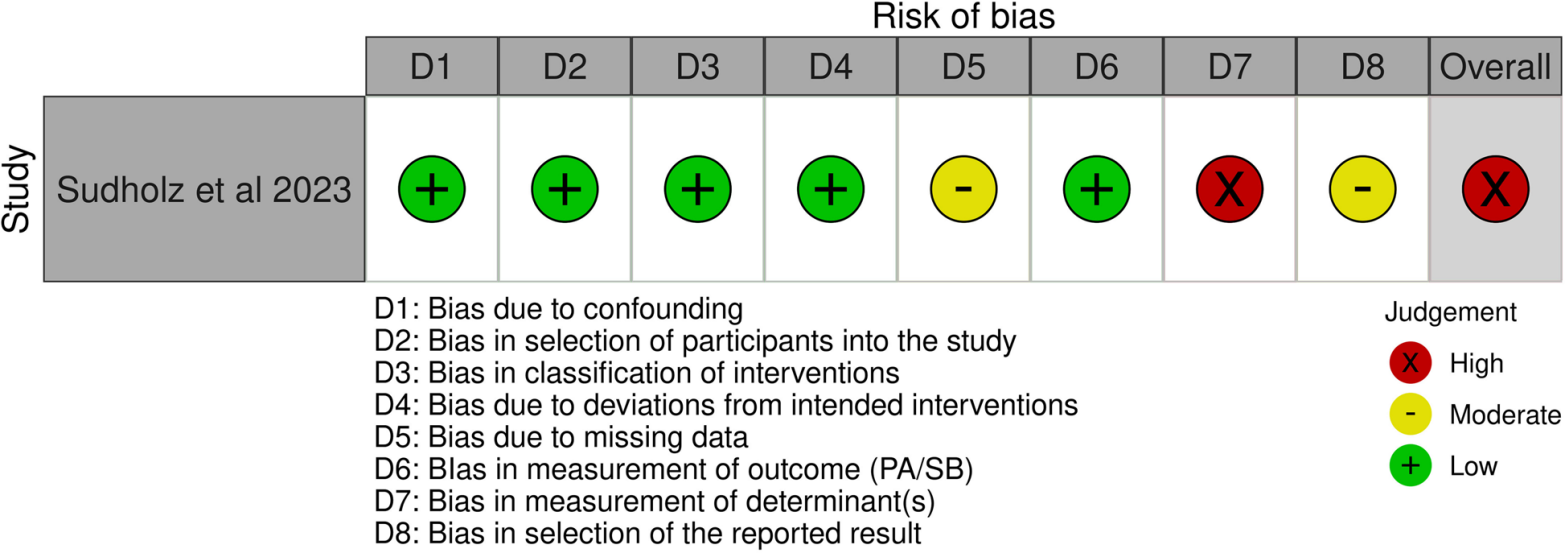
Risk of bias assessments of CT in the school setting

The results of the risk of bias assessment are presented in the traffic light plot [68] in Fig. 4. An overall high-risk of bias evaluation was determined for the 10 RCTs in the school setting. Domain 5 (bias due to measurement of determinants) and domain 2 (bias due to deviations from the intended interventions) mainly contributed to these evaluations. Regarding domain 5, participants were unlikely to be blinded in most interventions involving self-report measurements of the determinants. For domain 2, the lack of an appropriate analysis used to estimate the effect of assignment to intervention and the potential impact of this failure on the result contributed to these evaluations. An overall high-risk was assessed for the one CT in the school setting mainly due to the judgement in domain 7 (bias due to measurement of determinants), domain 5 (bias due to missing data) and domain 8 (bias in selection of reported results) (Fig. 5).

#### Certainty of the evidence

The certainty of the evidence for PA, SB, PA (follow-up), friendship quality, basic psychological needs, self-efficacy, social support by peers, perceived barriers to PA, and enjoyment was rated as low and for intentions, autonomous motivation, controlled motivation, and perceived autonomy support as very low, largely due to imprecision (Table 3).

**Table 3:** Quality of evidence (GRADE) of PA, SB, and modifiable determinants in settings

Outcome	Studies	(1)	(2)	(3)	(4)	(5)	Effect (95%CI)	Certainty
**School setting**								
PA	Andruschko et al. 2018 [59]; Casado-Robles et al. 2022 [65]; Corder et al. 2016 [53]; Corder et al. 2020 [54]; Ha et al. 2020 [55]; Hankonen et al. 2017 [60]; Jago et al. 2021 [56]; Schnider et al. 2021 [58]; Verswijveren et al. 2022 [66]	serious^a^	not serious	not serious	serious^e^	none	-0.04 (-0.18, 0.10)	⨁⨁◯◯ Low
SB	Andruschko et al. 2018 [59]; Casado-Robles et al. 2022 [65]; Corder et al. 2020 [54]; Hankonen et al. 2017 [60]; Jago et al. 2021 [56]; Verswijveren et al. 2022 [66]	serious^a^	serious^b^	not serious	serious^e^	none	-0.09 (-0.29, 0.11)	⨁⨁◯◯ Low
PA (follow-up)	Schnider et al. 2021 [58]; Verswijveren et al. 2022 [66]	serious^a^	not serious	not serious	serious^e^	none	-0.22 (-0.77, 0.23)	⨁⨁◯◯ Low
Friendship quality	Corder et al. 2016 [53]; Corder et al. 2020 [54]	serious^a^	not serious	not serious	serious^e^	none	0.08 (-0.35, 0.44)	⨁⨁◯◯ Low
Intentions	Ha et al. 2020 [55]; Casado-Robles et al. 2022 [65]; Schnider et al. 2021 [58]	serious^a^	serious^c^	not serious	serious^e^	none	-0.03 (-0.45, 0.33)	⨁◯◯◯ Very low
Autonomous motivation	Casado-Robles et al. 2022 [65]; Ha et al. 2020 [55]; Jago et al. 2021 [56]; Schnider et al. 2021 [58]	serious^a^	serious^c^	not serious	serious^e^	none	-0.06 (-0.41, 0.24)	⨁◯◯◯ Very low
Controlled motivation	Casado-Robles et al. 2022 [65]; Ha et al. 2020 [55]; Jago et al. 2021 [56]; Schnider et al. 2021 [58]	serious^a^	serious^c^	not serious	serious^e^	none	-0.10 (-0.49, 0.26)	⨁◯◯◯ Very low
Basic psychological needs	Ha et al. 2020 [55]; Jago et al. 2021 [56]	serious^a^	not serious	not serious	serious^e^	none	-0.01 (-0.34, 0.28)	⨁⨁◯◯ Low
Self-efficacy	Corder et al. 2016 [53]; Corder et al. 2020 [54]; Jago et al. 2021 [56]; Schnider et al. 2021 [58]; Verswijveren et al. 2022 [66]	serious^a^	not serious	not serious	serious^e^	none	0.05 (-0.09, 0.18)	⨁⨁◯◯ Low
Social support by peers	Corder et al. 2016 [53]; Corder et al. 2020 [54]; Jago et al. 2021 [56]	serious^a^	not serious	not serious	serious^e^	none	0.04 (-0.25, 0.30)	⨁⨁◯◯ Low
Perceived autonomy support	Casado-Robles et al. 2022 [65]; Ha et al. 2020 [55]	serious^a^	very serious^c^	not serious	very serious^f^	none	0.18 (-0.95, 1.07)	⨁◯◯◯ Very low
Perceived barriers to PA	Corder et al. 2016 [53]; Verswijveren et al. 2022 [66]	serious^a^	not serious	not serious	serious^e^	none	0.08 (-0.55, 0.52)	⨁⨁◯◯ Low
Enjoyment	Andruschko et al. 2018 [59]; Verswijveren et al. 2022 [66]	serious^a^	not serious	not serious	serious^e^	none	0.05 (-0.55, 0.56)	⨁⨁◯◯ Low
**School and family setting**								
PA	Aittasalo et al. 2019 [61]; Dewar et al. 2014 [62]; Lubans et al. 2010 [63]	serious^a^	serious^b^	not serious	serious^e^	none	0.05 (-0.86, 0.74)	⨁◯◯◯ Very low
Self-efficacy	Dewar et al. 2014 [62]; Lubans et al. 2010 [63]	serious^a^	serious^b^	not serious	serious^e^	none	0.04 (-0.39, 0.40)	⨁◯◯◯ Very low
Social support by family	Dewar et al. 2014 [62]; Lubans et al. 2010 [63]	serious^a^	not serious	not serious	serious^e^	none	-0.03 (-0.45, 0.31)	⨁⨁◯◯ Low

As the domain 5 of the risk of bias (i.e., risk of bias in measurement of the determinants) is almost inevitable in the nature of the interventions conducted, it was decided that it should be treated more leniently in GRADE

(1) = Risk of bias, (2) = Inconsistency, (3) = Indirectness, (4) = Imprecision, (5) = Other considerations

^a^Downgraded one level due to high and/or some concerns of bias in more than one domain for the majority of included studies in the respective outcome (see Figs. 4 and 7)

**Fig. 6:**
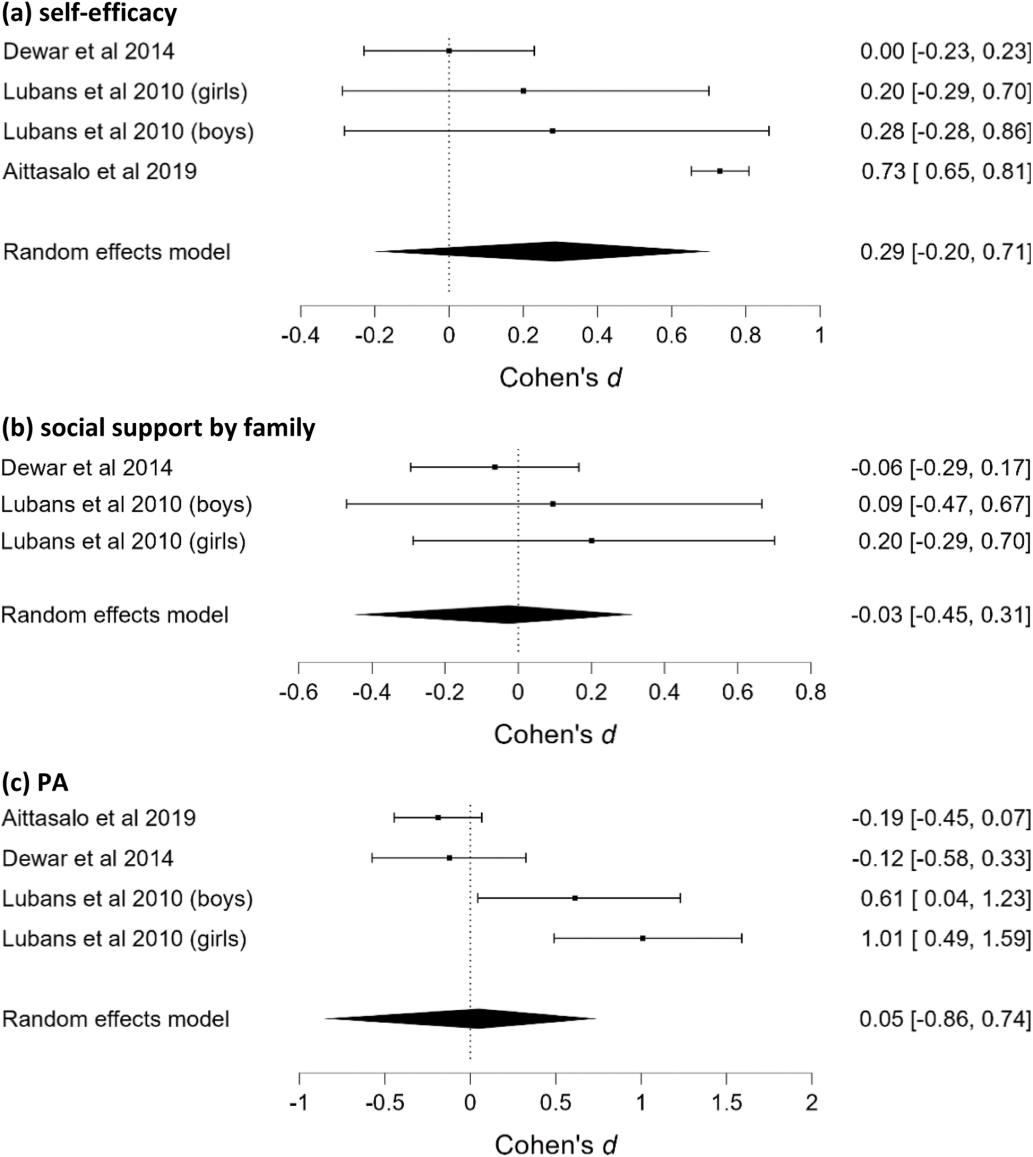
Forest plots of effects of interventions on (**a**) self-efficacy, (**b**) social support by family (**c**) PA in the school and family setting

^b^Downgraded one level due to inconsistency [minimal overlap of 95%CI (see respective forest plots in Figs. 3b, 6c, a) and evidence for heterogeneity]

^c^Downgraded one level due to inconsistency (lack of overlap of 95%CI; see respective forest plots in Fig. 2b,c, d)

^d^Downgraded two level due to inconsistency (lack of overlap of 95%CI and very different estimates; see respective forest plots in Fig. 2h)

^e^Downgraded one level due to imprecision (relatively wide 95%CI on the overall estimate including potential for both positive and negative effects; see respective forest plots in Figs. 2a-g, j, k, 3a, b, 6a-c)

^f^Downgraded two levels due to imprecision (very wide 95%CI on the overall estimate including potential for both positive and negative effects; see respective forest plot in Fig. 2h

#### School and family setting

Three RCTs [61–63], all theory-based, measuring habitual PA using accelerometers [61, 62] or pedometers [63], published from 2010 to 2019 were included. These studies included a school-based intervention with additional intervention components involving parents and PA related activities at home. The number of the participants ranged from 124 to 1550 and the intervention duration ranged from four weeks to one year. Lubans et al. [63] analyzed data separately for boys and girls and thus this study was introduced in the MA twice, once for boys and once for girls. No study included a post-intervention follow-up measure. Interventions focused mainly on increasing adolescents’ PA and/or decreasing SB and enhancing related psychosocial variables (Table 1).

#### Determinants

In total, 18 modifiable determinants were identified in the school and family setting (10 individual–psychological, one individual–behavioural, five interpersonal, and two institutional). Merging conceptually-related determinants into broader categories resulted in a final number of 10 determinants (five individual–psychological, one individual–behavioural, three interpersonal, and one institutional). Two of these determinants (i.e., self-efficacy and social support by family; Fig. 6a,b respectively) were measured in more than two studies and meta-analyzed. Moderate evidence to suggest the absence of an effect on self-efficacy and social support by family were found. The evidence to suggest the presence or absence of publication bias was insufficient (Table 4).

**Table 4:** Results of RoBMAs in the school and family setting for PA and determinants and the associated heterogeneity and publication bias assessments

	**n**	**Effect size estimates (95%CI)**	**BF**_**10**_
**MA – Self-efficacy** (Fig. 6a)	3	0.04 (-0.39, 0.40)	0.18*
Heterogeneity (τ)		0.14 (0.03, 0.43)	-
Publication bias		-	0.70
**MA – Social support by family** (Fig. 6b)	3	-0.03 (-0.45, 0.31)	0,15*
Heterogeneity (τ)		0.04 (0.00, 0.27)	-
Publication bias		-	0.55
**MA—PA** (Fig. 6c)	4	0.05 (-0.86, 0.74)	0.39
Heterogeneity (τ)		0.36 (0.00, 1.06)	-
Publication bias		-	1.68

*moderate evidence for absence of an effect

Regarding determinants measured in only one study, nonsignificant intervention effects (*d*s ranged from -0.04 to 0.15) were found.

#### PA

One RoBMA was conducted for PA (Fig. 6c) showing insufficient evidence to suggest the presence or absence of an effect on PA, publication bias and heterogeneity. (Table 4). Only one study [62] measured SB and reported negligible intervention effects (*d* = -0.08).

#### Risk of bias assessment

**Fig. 7:**
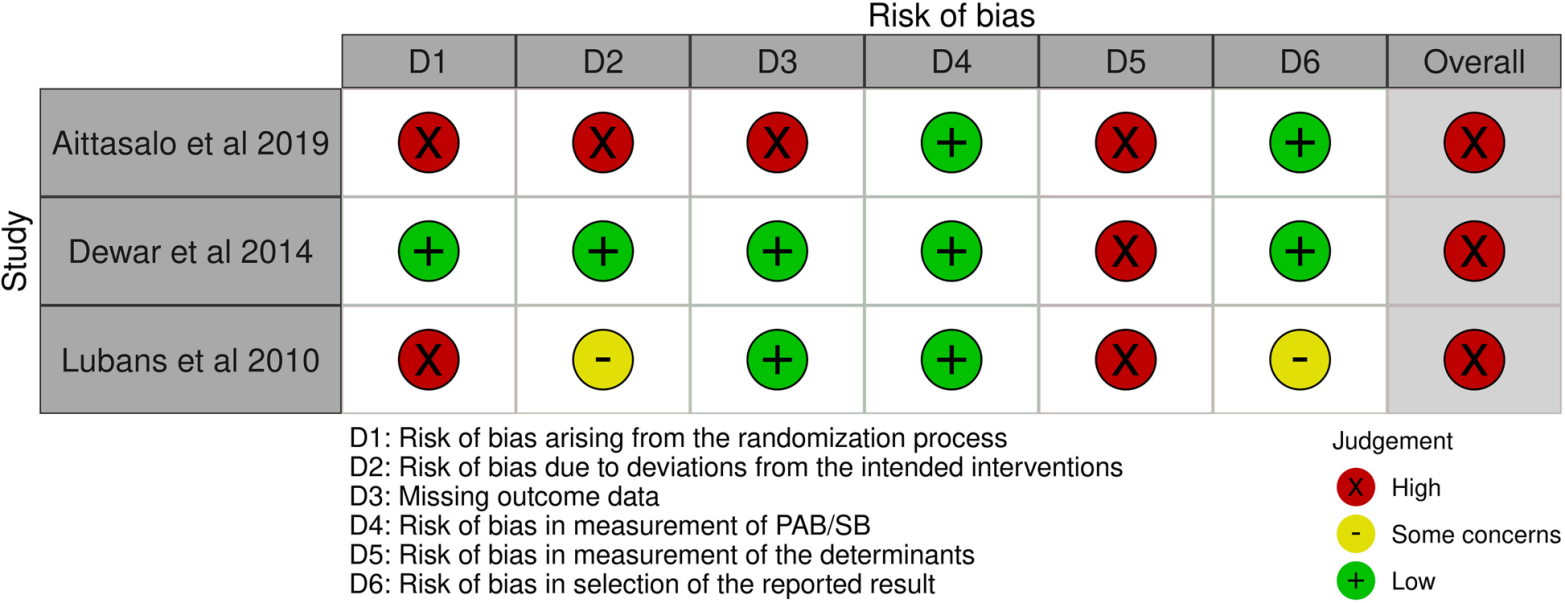
Risk of bias assessments of RCTs in the school and family setting

An overall high-risk of bias evaluation was determined for the three RCTs in the school and family setting. Domain 1 (bias arising from the randomization process), domain 5 (bias due to the measurement of determinants) and domain 2 (bias due to deviations from the intended interventions) primarily contributed to these evaluations (Fig. 7).

#### Certainty of evidence

The certainty of the evidence for PA and self-efficacy was rated as very low and for social support by family as low, largely due to imprecision (Table 3).

#### Family setting

One RCT [64] measuring habitual PA using a mobile phone-based pedometer app in the family setting was identified. Forty-two adolescent girls participated in this six-week multi-component intervention based on the self-determination theory. No follow-up measures were included (Table 1). The nine determinants (all individual–psychological) identified were merged into six broader determinants. Nonsignificant intervention effects on the determinants were found (*d*s ranged from -0.23 to 0.59). Notably, medium standardized mean differences in favour of the experimental group were found for body appreciation, *d* = 0.59, 95%CI (-0.03, 1.21), and small differences for perceived competence, *d* = 0.37, 95%CI (-0.24, 0.98), autonomous motivation, *d* = 0.33, 95%CI (-0.08, 0.73), and amotivation, *d* = 0.27, 95%CI (-0.34, 0.88). However, for all these determinants the 95%CIs crossed the threshold. No intervention effect on PA (*d* = 0.00) was found.

### Risk of bias assessment

**Fig. 8:**
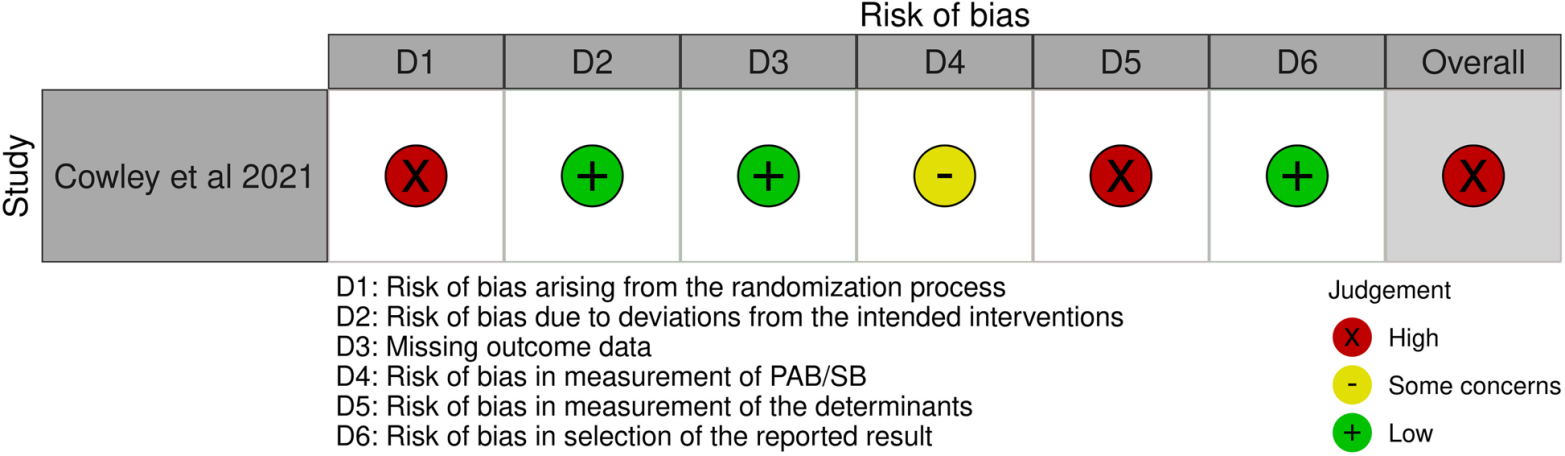
Risk of bias assessments of RCT in the family setting

An overall high-risk of bias evaluation was determined for this study [64] mainly due to domain 1 (bias arising from the randomization process) and domain 5 (bias due to the measurement of determinants) (Fig. 8).

#### Sensitivity analysis

For outcomes requiring estimations of composite scores, sensitivity analyses were performed showing no difference in the effect sizes when *r* was set at 0.2, 0.5 and 0.8.

## Discussion

The aim of this SRMA was threefold: (a) to identify modifiable determinants of adolescents’ device-based PA/SB that were targeted in RCTs and CTs in different settings, (b) to examine the intervention effects on PA/SB and modifiable determinants and (c) to investigate the potential associations of modifiable determinants with adolescents’ PA/SB. A wide range of modifiable determinants were identified. Generally, none or negligible evidence for intervention effect on adolescents’ device-based PA/SB and modifiable determinants were found, while the certainty of this evidence ranged from very low to low. Moreover, this review provided weak evidence regarding how modifiable the identified determinants are and the corresponding changes of these determinants with adolescents’ device-based PA/SB in three different settings (i.e., school, family, school and family). Methodological issues in the implementation and evaluation of the interventions were identified and the characteristics of the effective PA interventions were highlighted.

In particular, 54 determinants merged into 33 broader determinants were identified in the 14 RCTs and one CT included in this SRMA. Similar to previous reports [27], most of these determinants were individual–psychological, some interpersonal, and very few individual–behavioural or institutional [18]. None or negligible pooled intervention effects on the determinants were found. In many cases, RoBMAs provided insufficient evidence for the presence or the absence of an effect. Regarding the determinants identified in only one study, moderate to large differences in favour of the experimental group were found in knowledge of the environment for practicing PA, BCTs, and choice provided (school setting). However, in almost all cases, these differences did not correspond to respective improvements in PA. Moreover, none or negligible pooled intervention effects on adolescents’ device-based PA or SB in settings were found. Intervention effects on reducing SB were found only in the CT [67] examining sitting in the classroom. Post intervention short-term and long-term follow-up effects on PA/SB were also negative. The lack of changes in the identified determinants and the corresponding PA/SB may be due to ineffectiveness of the interventions. This, however, does not imply the lack of relationships between determinants and PA/SB. Actually, considering that determinants have been viewed as “causal factors, and variations in these factors are followed systematically by variations in PA” ([17], p. 6), the lack of change in determinants found in the present review was rather expected to lead to lack of change in PA/SB.

Previous studies have also reported minimal effects of interventions on adolescents’ device-based PA [8]. Indeed, a SRMA of RCTs in school settings found small and nonsignificant pooled effects on device-based measured total PA and MVPA [69, 70]. Similarly, small to negligible pooled intervention effects on device-based PA and a small effect on MVPA in RCTs or CTs with participants younger than 16 years old were found [71, 72]. Other reviews including studies measuring both self-report and device-based PA found a very small positive effect of school-based PA intervention on adolescent girls’ PA [73], negligible effects on MVPA and no effects on total PA in children aged 5 to 18 years [74].

Within the school setting, evidence for the absence of an effect was found on both determinants (i.e., basic psychological needs, self-efficacy, friendship quality, intentions, autonomous and controlled motivation, social support by peers, and perceived autonomy support) and PA/SB. Available evidence regarding these determinants is mixed, whereas for some determinants only evidence from studies with adults were available. For example, a recent SRMA [75] has suggested, with low certainty of evidence, that school-based PA interventions may be effective in increasing some motivational outcomes (i.e., autonomous forms of motivation and task orientation) but less so for others (i.e., basic psychological needs). A MA of cross-sectional or longitudinal studies suggested that social support was not a strong predictor of adolescent girls’ PA [76], while SRMAs of longitudinal and experimental [77] and RCT studies [78] in adults reported positive associations between intentions and PA levels.

Narratively, large differences in favour of the experimental group were found for knowledge of the environment for practicing PA [65] without, however, corresponding effects on PA or SB. Knowledge about practicing PA may be a facilitator of adolescents’ PA [34]. Similarly, large effects were also found for BCTs (e.g., goal setting and self-monitoring) [60] without corresponding effect on PA. The evidence on the BCTs like goal setting, and self-monitoring is limited, and although there is evidence of promise, as highlighted from the feasibility study [60], the evidence is not sufficiently robust, and should be treated with caution. Following some positive associations between PA and self-monitoring, goal setting, and other self-regulatory BCTs reported in various settings among adults [79], the effects of these determinants on adolescents’ PA may be further explored. Moderate intervention effects on students’ perceptions of choice provided by their teacher but not on MVPA and SB after a single 20-min physical education session were found [57]. This finding was aligned with previous evidence where teachers’ perceived autonomy support did not increase students’ PA [80]. Generally, interventions can increase students’ MVPA during physical education classes [81], although, considering the results of the present and previous studies [73], the effects of school-based interventions on increasing students’ total PA cannot be ascertained.

In the school and family setting, RobMAs revealed insufficient evidence to suggest the presence or absence of an effect on PA and moderate evidence to suggest absence of an effect on self-efficacy and social support by family. Negligible, pooled intervention effects were found for self-efficacy and PA with evidence for heterogeneity. Yet, through a mediation study [63] the positive intervention effects on self-efficacy were not associated with changes in PA. Convincing evidence regarding the positive associations between self-efficacy and overall PA in adolescents [27] and the reduction of SB in both children/adolescents and adults [82] have been previously reported. Regarding social support, previous evidence suggests that it is not a strong predictor of PA in adolescent girls [76]. Narratively, positive intervention effects on family norm of setting limitations for screen time were detected but no corresponding effects on the device-based PA were found [61]. Aittasalo et al. [61], however, used non-validated questionnaires for measuring parental indicators and faced a high dropout rate from the accelerometer both pre- and post-intervention.

Only one RCT [64] identified in the family setting showing nonsignificant intervention effects on the determinants (all individual–psychological) and PA. Previous evidence has suggested that perceived competence was positively associated with PA when mainly measured through self-report methods among children and adolescents [83]. Regarding motivation, although weak to moderate positive associations of PA with autonomous forms of motivation in children and adolescents have been reported [84], a more recent SRMA using a meta-regression analysis showed that increases in autonomous motivation were not significantly related to increases in PA [79]. Thus, further research should explore the effects of the determinants on adolescents’ PA/SB in family setting, including determinants related to parents and family environment.

The certainty of evidence found ranged from very low to low mainly due to the high risk of bias and imprecision (i.e., relatively wide 95%CI on the overall estimate including the potential for both positive and negative effects). Possible explanations for the lack of intervention effects on PA/SB and determinants may include poor implementation of the interventions [54, 63], use of non-validated questionnaires for measuring the determinants [60, 61], use of scales with low measurement sensitivity or cultural appropriateness [63], and with dropout rates ranging from 22 to 83% [56, 58, 61, 65]. Some interventions included samples of 20 to 42 participants [59, 60, 64] or a small number of sessions ranging from 1 to 8 [55, 57, 58, 61], during which effects on determinants and consequently on PA/SB might not be feasible. Changing determinants may require longer time to occur. Some studies [53, 54, 56, 62] failed to comply with the suggested minimum period of four to five days of monitoring for capturing valid and reliable habitual PA [85], while others did not report the minimum accelerometer wear-time [59, 63]. Similar interpretations have been previously reported including issues related to the duration or the intensity of interventions, the quality of implementation, or the lack of checking implementation fidelity including the sound translation of theory-based principles into practical tasks and activities involved in interventions components [71, 73, 74]. Undoubtedly, all these issues represent challenges to be overcome in future research focusing on implementing well-designed interventions for examining which determinants are modifiable in settings and their levels of association with PA/SB.

Thus, the questions about the characteristics of the effective PA interventions and which of the determinants of adolescents’ PA/SB are modifiable are still open. Current interventions to promote adolescents’ PA are mostly ineffective [8, 62–74]. Considering the low levels of PA among adolescents [7] that reduced even more during COVID-19 lockdown [86], and the increased levels of SB which has been characterized as a global pandemic [87], the design and implementation of the most effective interventions is urgently needed. However, increasing PA is not an easy goal to reach by just focusing on simple, often short-term, individual health outcomes, or by involving isolated interventions but rather requires complex, multiple, innovative actions for creating active societies, environments, people, and systems [16]. In line with this, the International Society for Physical Activity and Health [16] has suggested that increasing PA and reducing SB requires multiple policies and actions across different settings including school, active transport, sport and recreation, and community. Considering that most adolescents spend a lot of their day-time in the school setting, a whole-of-school approach to PA should be a priority [13]. This involves the design of multicomponent interventions including as a cornerstone regular and high-quality physical education classes for all, suitable physical and social environments and resources, and policy actions to promote PA before, during, and after school hours (e.g., active recess breaks, active school transportation, extracurricular PA and sport, use of technology). For example, walking to and from school can have a meaningful contribution to adolescents’ PA [88], while using activity trackers may increase PA [89]. Thus, such approaches should be incorporated into respective interventions that may also be context-specific adopting a flexible approach that enables schools to tailor content to their specific context [90]. The involvement of staff, family and the wider community may also be necessary components of such interventions [8, 16]. Although, some evidence regarding the effectiveness of the multicomponent interventions has been reported [73, 91], further research is warranted to identify the most effective modifiable determinants of adolescents’ PA/SB, that these interventions should focus on in each setting [16]. Finally, close collaboration and establishment of a clear communication process between researchers, practitioners and policy and decision makers regarding the design, implementation, and evaluation of PA/SB interventions are also welcomed, as this would lead to increased effectiveness of PA/SB interventions in real-life contexts [92].

### Strengths and limitations

This study identified the modifiable determinants of adolescents’ PA/SB in settings and explored their associations with PA/SB. Including studies measuring concurrently PA/SB and modifiable determinants in specific settings provided the advantage of placing the factors (i.e., modifiable determinants) associated with adolescents’ PA in their social and environmental context [18]. Moreover, by focusing on high-quality studies (i.e., RCTs and CTs) potential causality between modifiable determinants and adolescents’ PA/SB could be detected [17]. Furthermore, to strengthen the quality of the evidence, RoBMA was the method of analysis allowing us to adjust for publication bias and enhance our understanding of the data by quantifying evidence on a continuous scale and assessing potential evidence for the null or the alternative hypothesis or whether results were inconclusive [46].

The relatively small number of studies included in MAs may be considered a limitation. This may be reflected in the results of some RoBMAs showing insufficient evidence to suggest the presence or the absence of an effect that may contribute to imprecision in the GRADE process. Moreover, the risk of bias assessment in the GRADE process was based on the evaluation of all domains and not only on the overall evaluation which was deemed high for all studies, because the lack of blinding of participants might have affected the measurement of determinants. Effective blinding of participants assigned to the intervention groups is a real challenge for future interventions. Heterogeneity was present in some MAs, while the evidence to suggest the presence or absence of publication bias was insufficient. Associations between PA/SB and modifiable determinants could only be inferred as almost all included studies did not conduct mediation analyses to test causal relationships. The use of different forms of PA/SB analyzed in MAs or the merge of conceptually-related determinants into broader determinants (e.g., autonomy, competence, and relatedness were merged into the broader determinant of basic psychological needs) may also be considered as limitations. However, we adopted this approach to avoid conducting a larger number of MAs with small number of studies for different forms of PA/SB or for every single determinant that may make the interpretation of the results more difficult.

### Future research

Considering that research up to date has primarily adopted an individual approach to behavioural change focusing mainly on individual–psychological determinants, future research may prioritize environmental over individual approaches for promoting PA [93] focusing on determinants related to physical environments, institutional, community, or policy factors [8, 18]. It is also critical for future studies to examine the mechanisms underlying behavioural changes related to adolescents’ PA/SB and conduct mediation analyses to explore these mechanisms [93]. In this direction, realist synthesis may enhance our understanding of what determinants of PA/SB might work, how they work, for who, and in what settings [94]. Further research should examine the effects of interventions in other settings in which adolescents are involved except that of family and school (e.g., sport and recreation, transportation, and community). For example, after school PA and sport programs can contribute to adolescents’ daily PA and reduced SB [95]. Most importantly, interventions involving more than one setting [16] adopting a transdisciplinary collaboration and targeting modifiable determinants from different categories [18] should further highlight the dynamic associations between determinants and settings and provide insightful information to guide related policies and practices. Considering that in the present study only three interventions involved follow-up measures, future research should explore the long-lasting intervention effects on adolescents’ PA/SB and modifiable determinants.

## Conclusions

This study found none or negligible evidence for intervention effects on adolescents’ device-based PA/SB and modifiable determinants in different settings. Some intervention effects in favour of the experimental group were found in single studies, for few determinants (i.e., knowledge for practicing PA, BCTs, choice provided). This evidence was rather limited and, in some cases, insufficient to draw a definite conclusion. Thus, the modifiable determinants of adolescents’ PA/SB should be further targeted in holistic multicomponent interventions in different settings and tested by well-designed, well-implemented and well-evaluated research.

### Supplementary Information

Supplementary Material 1.

Supplementary Material 2.

Supplementary Material 3.

## Data Availability

The data underlying the results presented in this review are available on request from the first author (akolov@pe.uth.gr).

## Abbreviations

BCTs: Behaviour Change Techniques
CI: Credible Interval
CT: Controlled Trials
DE-PASS: DEterminants of Physical Activities in SettingS
GRADE: Grading Recommendations to Assess Development and Evaluation system
MA: Meta-Analysis
MVPA: Moderate to Vigorous PA
PA: Physical Activity
PRISMA: Preferred Reporting Items for Systematic Reviews and Meta-Analyses
RCT: Randomized Control Trial
RoBMA: Robust Bayesian Meta-Analyses
SB: Sedentary Behaviour
SR: Systematic Review
SRMA: Systematic Review and Meta-Analysis
WHO: World Health Organization
