# Supplementary material for: DE-PASS best evidence statement (BESt): determinants of adolescents’ device-based physical activity and sedentary behaviour in settings: a systematic review and meta-analysis
Source: BMC Public Health. 2024 Jun 26;24:1706. doi: 10.1186/s12889-024-19136-y (PMC11202347; doi:10.1186/s12889-024-19136-y)
Supplement: Supplementary file 1 — Supplementary Material 1. [file 12889_2024_19136_MOESM1_ESM.docx]

**Additional file 1**

**Composite score and effect size calculation**

**1a. Composite score calculation for multiple outcomes (Borenstein et al., 2021)**

Formula for calculating composite score:

$$var\left( \sum_{a=1}^{m} Y_{a} \right)= \sum_{a=1}^{m} V_{a}+ \sum_{a\neq b} \left( r_{ab}\sqrt{V_{a}} \sqrt{V_{b}} \right)$$

where *Y* is the effect size of an outcome, *V* is the variance of *Y* for several variables *a*=1…m, and *r* is the correlation coefficient that indicates the extent to which the outcomes co-vary.

**1b. Effect size calculation**

***Continuous outcomes***

When within group change scores (SD) were reported in the primary study, these were used to calculate standardized mean difference (SMD). If measures of variance other than SD were reported in each group, e.g., 95%CI or standard error (SE), these were converted to SD as reported in Cochrane handbook (<https://training.cochrane.org/handbook/current/chapter-06#section-6-5-2-2>).

SMD was then calculated using the Cohen’s d formula:

$$SMD=\frac{\mu_{1}-\mu_{2}}{\sigma_{pooled}}$$

$$\sigma_{pooled}=\sqrt{\frac{{(n}_{1}-1)\sigma_{1}^{2}+ {(n}_{2}-1)\sigma_{2}^{2}}{{(n}_{1}+ n_{2}-2)}}$$

Standard error (SE) of SMD was calculated as:

$${SE}_{SMD}=\sqrt{\frac{n_{1}+ n_{2}}{n_{1}n_{2}}+\frac{{SMD}^{2}}{2(n_{1}+ n_{2}-2)}}$$

In cases where:

1. within group change scores (SD) were not reported, pre- and post- SD were transformed to SD change as reported in Cochrane handbook (<https://training.cochrane.org/handbook/current/chapter-06#section-6-5-2-8>). Correlation coefficients were arbitrarily set at 0.5 when this information was not provided in the study;
2. only between-group differences were reported with t statistics or *p*-values, these were converted to SD as reported in Cochrane handbook (<https://training.cochrane.org/handbook/current/chapter-06#section-6-5-2-3>).
