# Supplementary material for: DE-PASS best evidence statement (BESt): determinants of adolescents’ device-based physical activity and sedentary behaviour in settings: a systematic review and meta-analysis
Source: BMC Public Health. 2024 Jun 26;24:1706. doi: 10.1186/s12889-024-19136-y (PMC11202347; doi:10.1186/s12889-024-19136-y)
Supplement: Supplementary file 2 — Supplementary Material 2. [file 12889_2024_19136_MOESM2_ESM.docx]

**Additional file 2**

**Interpretation of Bayes factors (BF_10_) as evidence for null hypothesis and alternative hypothesis***

| **BF_10_** | **Description** |
| --- | --- |
| >100 | Extreme evidence for alternative hypothesis |
| 30 – 100 | Very strong evidence for alternative hypothesis |
| 10 – 30 | Strong evidence for alternative hypothesis |
| 3 – 10 | Moderate evidence for alternative hypothesis |
| 1 – 3 | Anecdotal evidence for alternative hypothesis |
| 1 | No evidence |
| 1– 1/3 (0.33) | Anecdotal evidence for null hypothesis |
| 1/3 (0.33) – 1/10 (0.1) | Moderate evidence for null hypothesis |
| 1/10 (0.1) – 1/30 (0.033) | Strong evidence for null hypothesis |
| 1/30 (0.033) – 1/100 (0.01) | Very strong evidence for null hypothesis |
| < 1/100 (< 0.01) | Extreme evidence for null hypothesis |
| *Note*: BF_10_ represents the Bayes factors of the alternative hypothesis (i.e., a treatment has some real effect) against the null hypothesis (i.e., the effect does not exist). | |
| *Lee MD, Wagenmakers E.J. Bayesian cognitive modeling: A practical course. In Bayesian cognitive modeling: A practical course; Cambridge University Press: Cambridge, UK, 2013. | |
