# Supplementary material for: DE-PASS best evidence statement (BESt): determinants of adolescents’ device-based physical activity and sedentary behaviour in settings: a systematic review and meta-analysis
Source: BMC Public Health. 2024 Jun 26;24:1706. doi: 10.1186/s12889-024-19136-y (PMC11202347; doi:10.1186/s12889-024-19136-y)
Supplement: Supplementary file 3 — Supplementary Material 3. [file 12889_2024_19136_MOESM3_ESM.docx]

**Additional file 3**

**Frequentist approach to the meta-analyses**

This supplementary file provides the results of the standard mean differences (SMD) with 95% confidence interval (CI) for the MAs conducted in this review. For effect size, we considered Cohen’s *d* ≥ 0.2 (small effect), ≥ 0.5 (moderate effect), ≥ 0.8 (large effect) (Cohen, 1988). For heterogeneity, we have reported *τ*² values. We have also reported *I^2^* values, whereby values of 25%, 50% and 75% indicates low, moderate and high inconsistency respectively^[[1]](#footnote-1)^. We were unable to test publication bias by funnel plot asymmetry inspection, as none of our MAs included the recommended ≥10 studies in an MA^[[2]](#footnote-2)^.

**School setting**


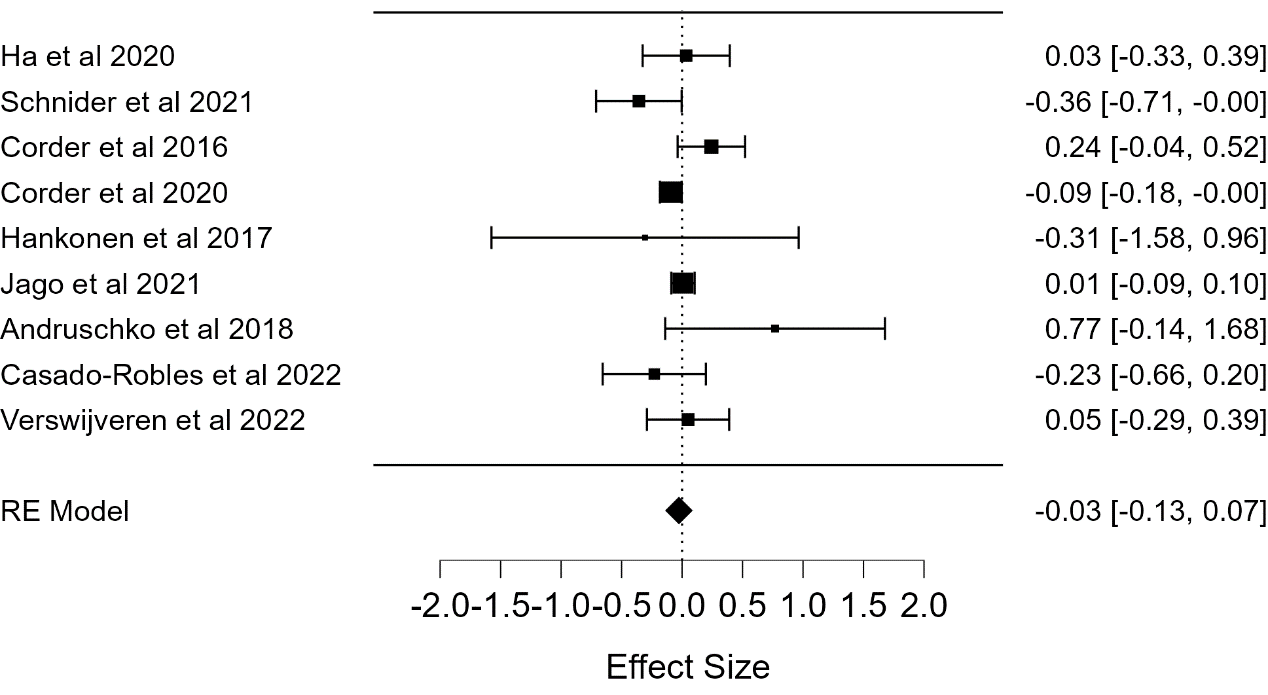


**Figure 1.** Forest plot showing intervention effects on PA in RCTs in school setting, *d* = -0.03, 95%CI (-0.13, 0.07), *τ*² = 0.006, *I^2^* = 33.55% (moderate inconsistency).


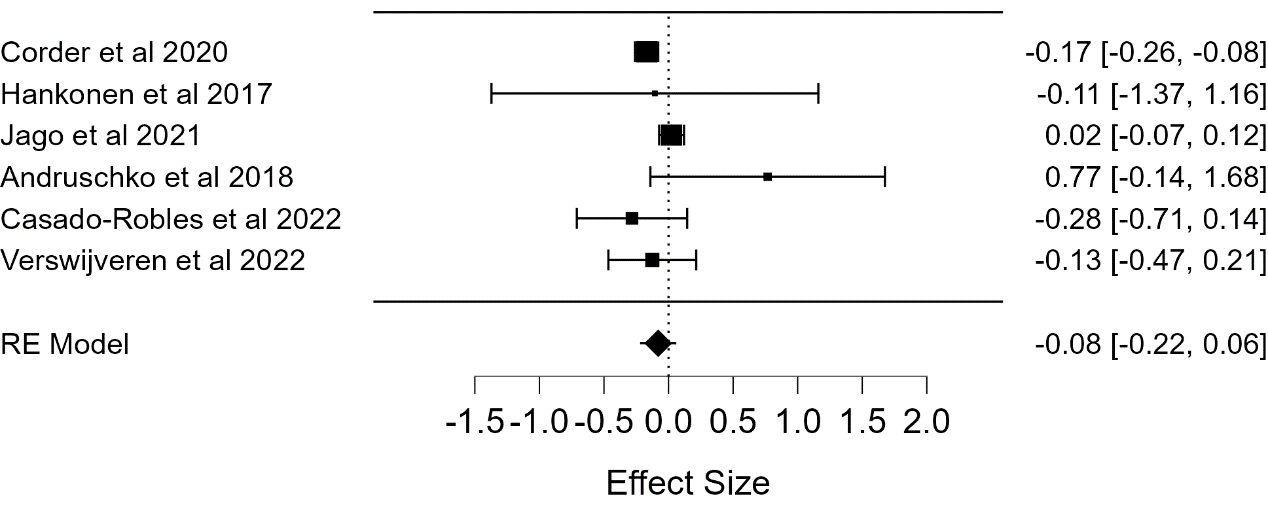


**Figure 2.** Forest plot showing intervention effects on SB in RCTs in school setting, *d* = -0.08, 95%CI (-0.22, 0.06), *τ*² = 0.011, *I^2^* = 53.12% (moderate inconsistency).


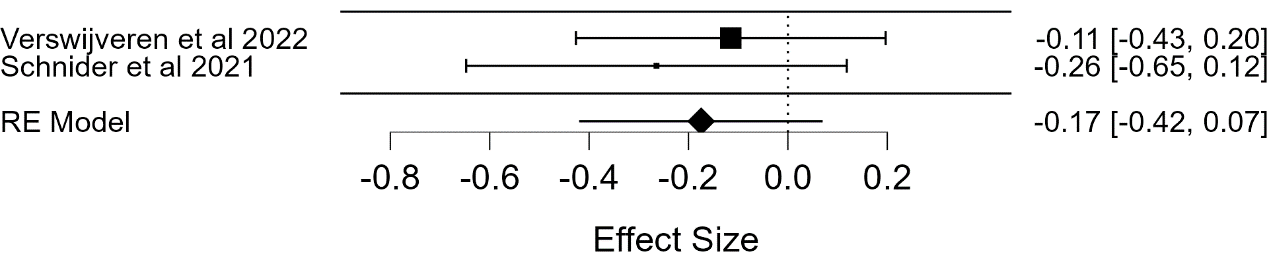


**Figure 3.** Forest plot showing intervention effects on short-term (up to six months) post-intervention follow-up PA in RCTs in school setting, *d* = -0.17, 95%CI (-0.42, 0.07), *τ*² = 0.000, *I^2^* = 0.00% (low inconsistency).


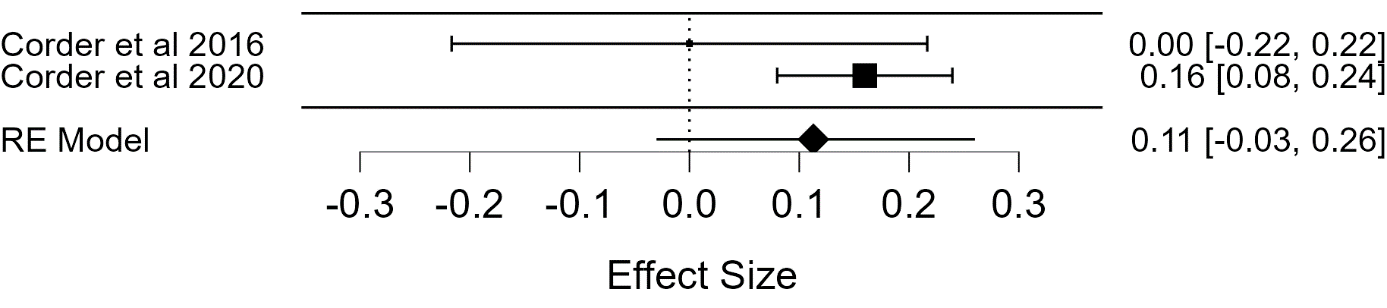


**Figure 4.** Forest plot showing intervention effects on friendship quality in RCTs in school setting, *d* = 0.11, 95%CI (-0.03, 0.26), *τ*² = 0.006, *I^2^* = 45.63% (moderate inconsistency).


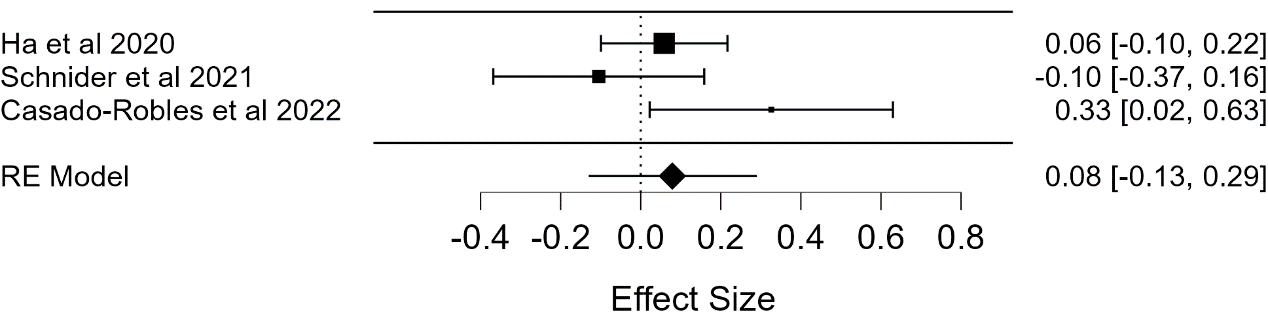


**Figure 5.** Forest plot showing intervention effects on intentions in RCTs in school setting, *d* = 0.08, 95%CI (-0.13, 0.29), *τ*² = 0.019, *I^2^* = 56.78% (moderate inconsistency).


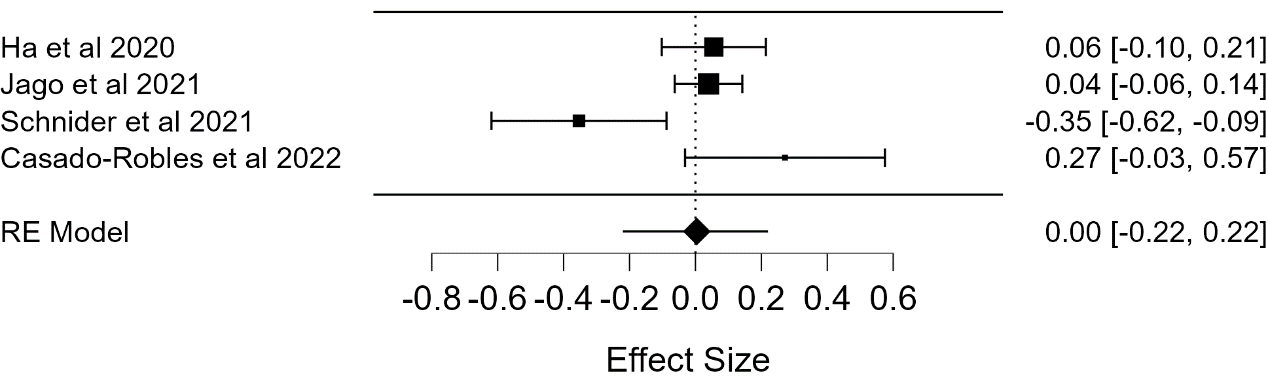


**Figure 6.** Forest plot showing intervention effects on autonomous motivation in RCTs in school setting, *d* = 0.00, 95%CI (-0.22, 0.22), *τ*² = 0.039, *I^2^* = 82.10% (high inconsistency).


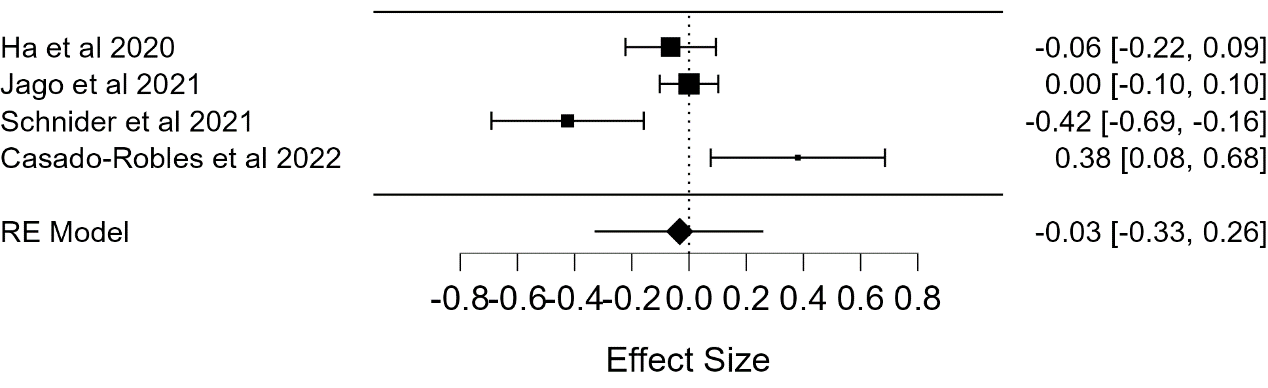


**Figure 7.** Forest plot showing intervention effects on controlled motivation in RCTs in school setting, *d* = -0.03, 95%CI (-0.33, 0.26), *τ*² = 0.078, *I^2^* = 90.16% (high inconsistency).


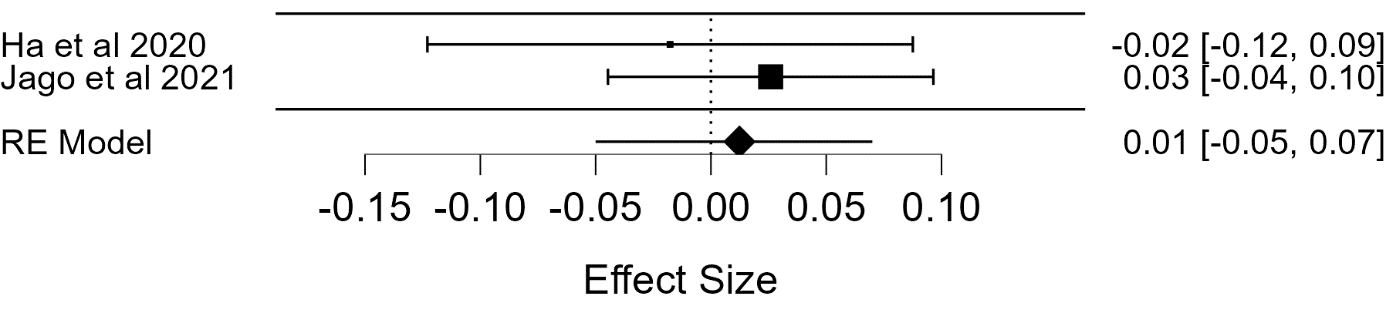


**Figure 8.** Forest plot showing intervention effects on basic psychological needs in RCTs in school setting, *d* = 0.01, 95%CI (-0.05, 0.07), *τ*² = 0.000, *I^2^* = 0.00% (low inconsistency).


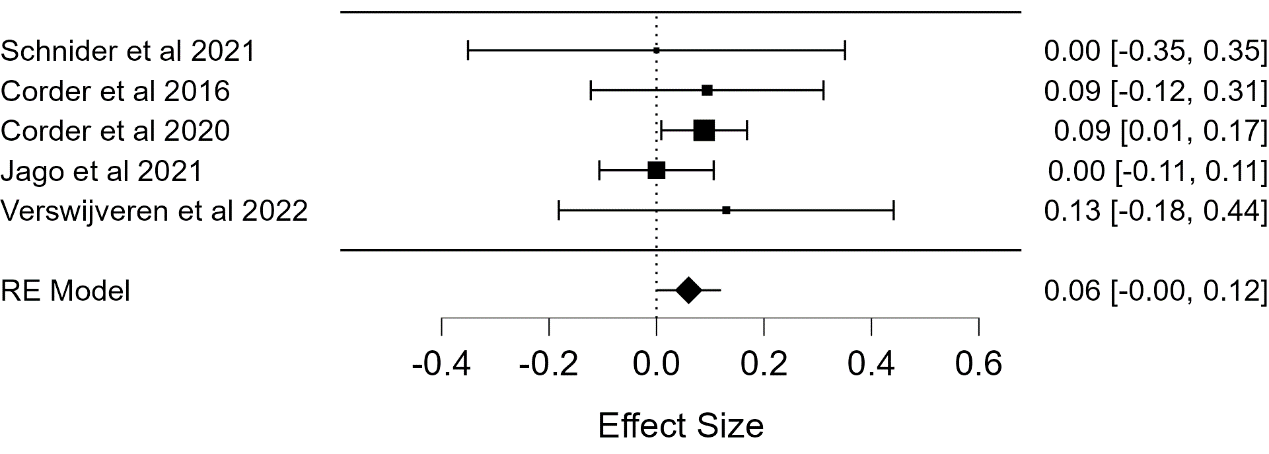


**Figure 9.** Forest plot showing intervention effects on self-efficacy in RCTs in school setting, *d* = 0.06, 95%CI (-0.00, 0.12), *τ*² = 2.296×10^-4^, *I^2^* = 3.59% (low inconsistency).


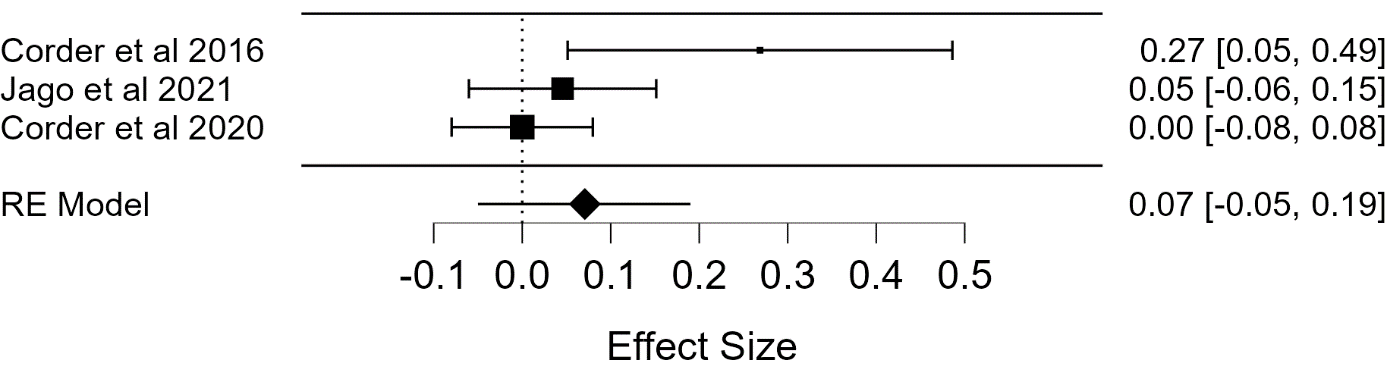


**Figure 10.** Forest plot showing intervention effects on social support by peers in RCTs in school setting, *d* = 0.07, 95%CI (-0.05, 0.19), *τ*² = 0.008, *I^2^* = 68.17% (moderate inconsistency).


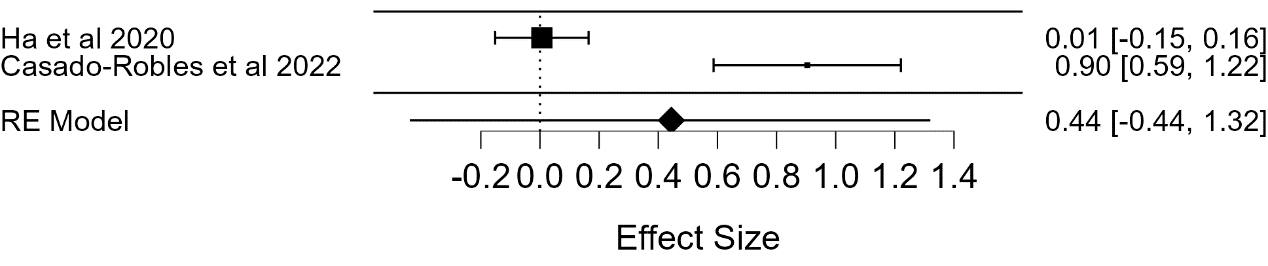


**Figure 11.** Forest plot showing intervention effects on perceived autonomy support in RCTs in school setting, *d* = 0.44, 95%CI (-0.44, 1.32), *τ*² = 0.386, *I^2^* = 95.96% (high inconsistency).


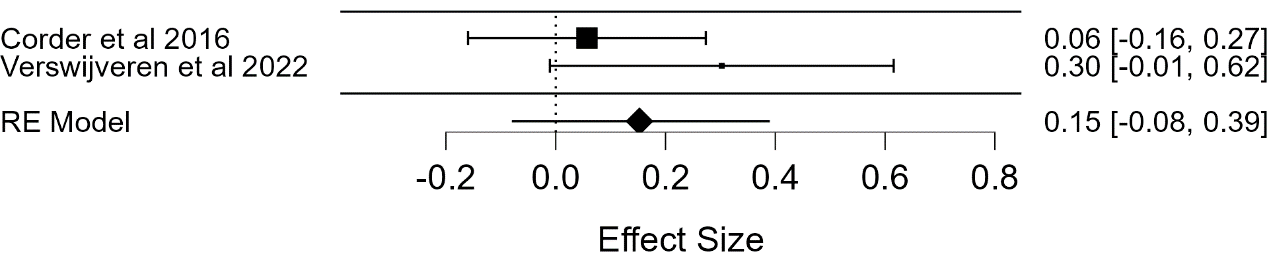


**Figure 12.** Forest plot showing intervention effects on perceived barriers to PA in RCTs in school setting, *d* = 0.15, 95%CI (-0.08, 0.39), *τ*² = 0.011, *I^2^* = 37.47% (moderate inconsistency).


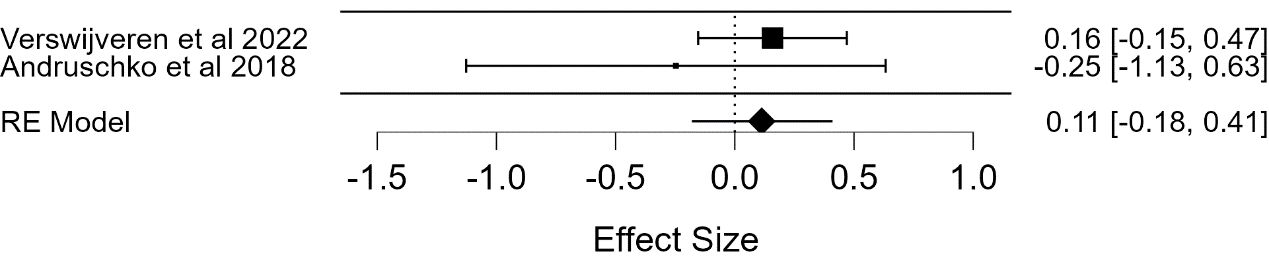


**Figure 13.** Forest plot showing intervention effects on enjoyment in RCTs in school setting, *d* = 0.11, 95%CI (-0.18, 0.41), *τ*² = 0.000, *I^2^* = 0.00% (low inconsistency).

**School and family setting**


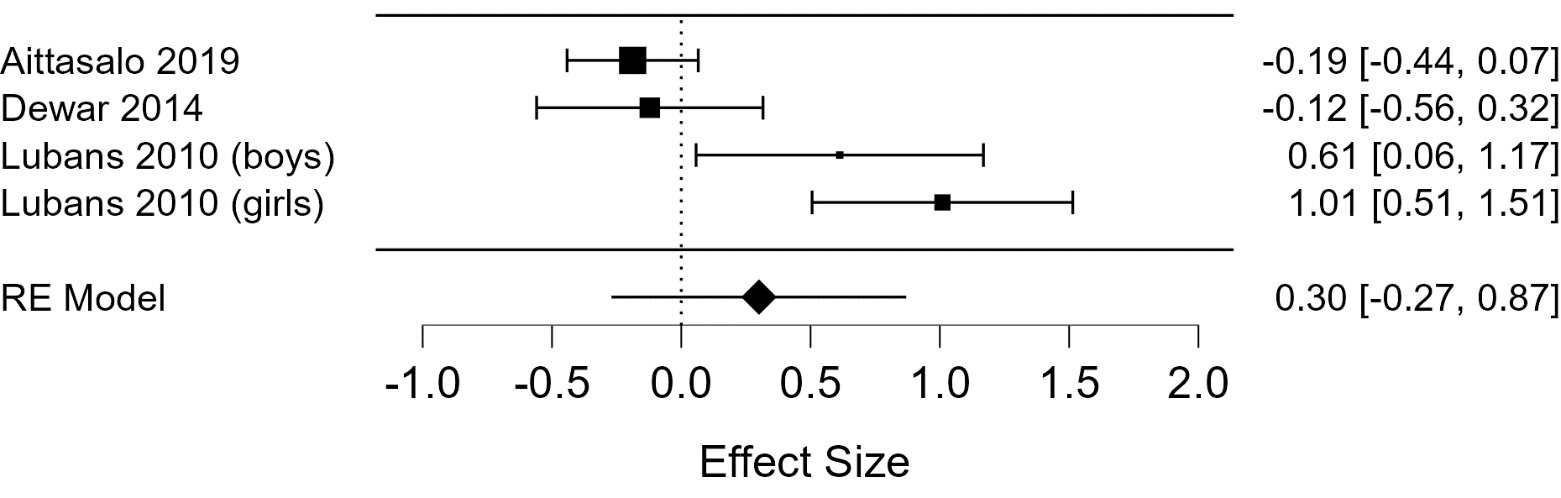


**Figure 14**. Forest plot showing intervention effects on PA in RCTs in the combined school and family setting, *d* = 0.30, 95%CI (-0.27, 0.87), *τ*² = 0.290, *I^2^* = 86.57% (high inconsistency).


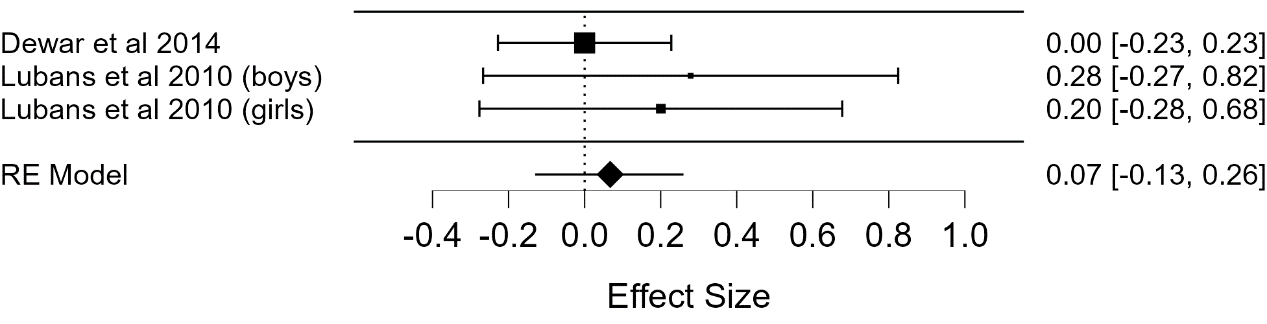


**Figure 15**. Forest plot showing intervention effects on self-efficacy in RCTs in the combined school and family setting, *d* = 0.33, 95%CI (-0.04, 0.69), *τ*² = 0.000, *I^2^* = 0.00% (low inconsistency).


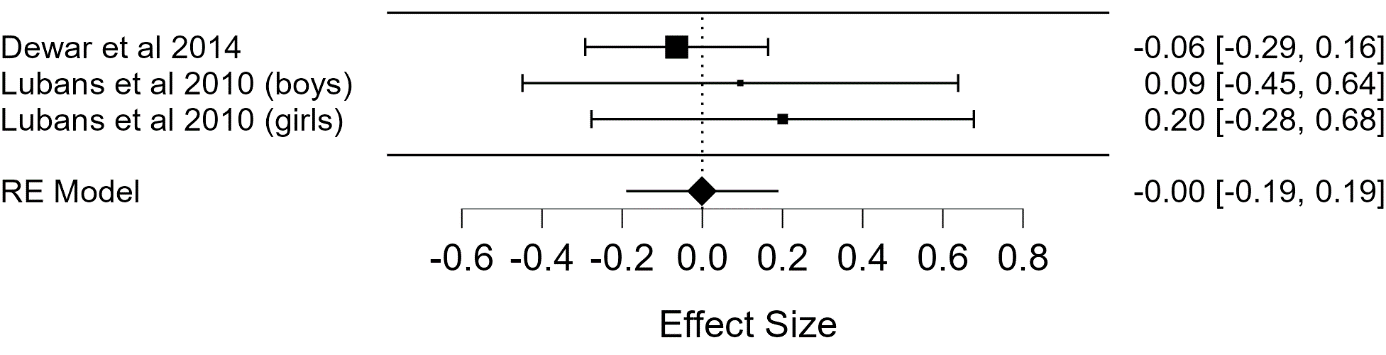
**Figure 16**. Forest plot showing intervention effects on social support by family in RCTs in the combined school and family setting, *d* = -0.00, 95%CI (-0.19, 0.19), *τ*² = 0.000, *I^2^* = 0.00% (low inconsistency).

1. Cochran’s Q was not used to assess heterogeneity as all MAs are under-powered due to the low number of studies in all MAs (Gavaghan et al., 2000).

   [Gavaghan DJ, Moore AR, McQay HJ. An evaluation of homogeneity tests in meta-analysis in pain using simulations of patient data. Pain. 2000;85:415-24](http://www.painjournalonline.com/article/S0304-3959(99)00302-4). [↑](#footnote-ref-1)
2. Sterne JAC, Sutton AJ, Ioannidis JPA, Terrin N, Jones DR, Lau J, Carpenter J, Rücker G, Harbord RM, Schmid CH, Tetzlaff J, Deeks J.J, Peters J, Macaskill P, Schwarzer G, Duval S, Altman DG, Moher D, Higgins JP.T. Recommendations for examining and interpreting funnel plot asymmetry in meta-analyses of randomised controlled trials. BMJ. 2011;343:d4002. [↑](#footnote-ref-2)
